# Activation of cryptic biosynthetic gene clusters by fungal artificial chromosomes to produce novel secondary metabolites

**DOI:** 10.3934/microbiol.2023039

**Published:** 2023-12-18

**Authors:** Chengcang C. Wu, Andrea A. Stierle, Donald B. Stierle, Hongyu Chen, Michael Swyers, Timothy Decker, Emili Borkowski, Peter Korajczyk, Rosa Ye, Niel Mondava

**Affiliations:** 1 Intact Genomics, Inc. 1100 Corporate Square Drive, Suite 257, St Louis, Missouri, 63132, USA; 2 Department of Biomedical and Pharmaceutical Sciences, University of Montana, Missoula, Montana 59812, USA

**Keywords:** Fungal artificial chromosome, next-gen-sequencing, cryptic biosynthesis, secondary metabolites, Nuclear Magnetic Resonance (NMR), Liquid Chromatography/Mass Spectrometry (LC/MS)

## Abstract

In 2017, we reported the discovery of Berkeleylactone A (BPLA), a novel, potent antibiotic produced exclusively in co-culture by two extremophilic fungi, *Penicillium fuscum* and *P. camembertii/clavigerum*, which were isolated from the Berkeley Pit, an acid mine waste lake, in Butte, Montana. Neither fungus synthesized BPLA when grown in axenic culture. Recent studies suggest that secondary metabolites (SMs) are often synthesized by enzymes encoded by co-localized genes that form “biosynthetic gene clusters” (BGCs), which might remain *silent* (inactive) under various fermentation conditions. Fungi may also harbor cryptic BGCs that are not associated with previously characterized molecules.

We turned to the tools of Fungal Artificial Chromosomes (FAC)-Next-Gen-Sequencing (NGS) to understand how co-culture activated cryptic biosynthesis of BPLA and several related berkeleylactones and to further investigate the true biosynthetic potential of these two fungi. FAC-NGS enables the capture of BGCs as individual FACs for heterologous expression in a modified strain of *Aspergillus nidulans* (heterologous host, FAC-*An*HH). With this methodology, we created ten BGC-FACs that yielded fourteen different SMs, including strobilurin, which was previously isolated exclusively from basidiomycetes. Eleven of these compounds were not detected in the extracts of the FAC-*An*HH. Of this discrete set, only the novel compound citreohybriddional had been isolated from either *Penicillium* sp. before and only at very low yield. We propose that through heterologous expression, FACs activated these silent BGCs, resulting in the synthesis of new natural products (NPs) with yields as high as 50%–60% of the crude organic extracts.

## 1. Introduction

SMs, also called natural products (NPs), have been an important source of new drugs and drug-like molecules for over 100 years [1],[2]. Since the 1990s major pharmaceutical companies have largely turned away from NP discovery efforts, citing difficulties in supply, screening, dereplication and characterization of these compounds relative to completely synthetic libraries [3].

Innovative approaches to NP drug discovery, however, have yielded promising results and renewed interest in the field. For example, fungi from extreme environments have proven to be a rich source of unique, bioactive compounds with drug-like potential. These include fungi that survive in temperatures as high as 90 °C or below 20 °C, in high salt environments and in extremes of pH [4]. We (Stierle lab) initiated a study of the secondary metabolites of the extremophilic fungi surviving in the Berkeley Pit, an acid mine waste lake in Butte, Montana. Enzyme inhibition assays targeting matrix metalloproteinase-3, caspase-1 and caspase-3 guided the isolation of compounds that blocked epithelial mesenchymal transition [5], inflammation [6],[7]; and apoptosis [8],[9] respectively. These efforts yielded a library of novel, bioactive compounds [10]–[19], that demonstrated the value of microbes from unstudied environments as a source of drug-like molecules.

Unfortunately, the need for more rapid identification of drug-like molecules has not been satisfied by traditional microbial drug discovery methods. Recent studies suggest that most secondary metabolites are synthesized by enzymes encoded by co-localized genes that form SM-BGCs that may remain silent when fungi are grown using standard growth conditions [20]. Efforts to elicit “cryptic biosynthesis” have shown that microbes can harbor BGCs that are not associated with previously characterized molecules [20],[21].

Several methods have been used to activate phenotypically silent SM-BGCs and to access potentially cryptic biosynthetic pathways in fungi. Changes in growth parameters including media composition, temperature, pH, duration, inclusion of specific enzyme inhibitors or promoters can have a dramatic effect on the secondary metabolite profile of particular fungi. Communal growth conditions can also activate silent genes [21]. Although most fungal secondary metabolite studies focus on fungi grown in pure culture, studies have shown that “crosstalk” between microorganisms can activate silent gene clusters and lead to the synthesis of novel secondary metabolites [22].

We explored the effects of fungal co-culture on the production of secondary metabolites of two extremophiles isolated from the Berkeley Pit: *Penicillium fuscum* and *P. camembertii/clavigerum* [23]. When grown in axenic culture, the most abundant compounds produced by *P. camembertii/clavigerum* were citrinin and patulin, and by *P. fuscum* was asperfuran. When grown in co-culture, however, these fungi produced two previously uncharacterized families of compounds that were not detectable in either axenic culture. These included the berkeleylactones, a novel family of fungal-macrolide antibiotics that exhibits potent activity against multi-drug resistant strains of *Staphylococcus aureus* and *Bacillus anthracis* [23].

Although these results were promising, the critical need for new drugs, especially antibiotics, requires a transformative approach to drug discovery. Scientists at Intact Genomics, Inc. and their collaborators invented FAC-NGS technology which can capture large unsequenced, random shear DNA fragments (up to 300 kb) and shuttle them into an engineered fungal host to produce FAC-transformants (FAC-Trs) capable of heterologous expression. This technology has been shown to result in the robust production of fungal SMs [24],[25]. It is proposed that this technology could yield a FAC-Tr capable of producing BPLA and other novel antibiotics in high yield.

While there are barriers to heterologous gene expression, rational refactoring methods have successfully introduced defined BGCs into a heterologous fungal host to induce production of novel SMs. For example, in an effort to find novel fungal meroterpenoids derived from 3,5-dimethylorsellinic acid (DMOA) in *Aspergillus insuetus*, Tang and Matsuda searched for DMOA synthase gene homologues in publicly available fungal genome databases and assessed the flanking regions of each identified gene [26]. They determined that *A. insuetus* CBS 107.25 contained a genomic region similar to the known gene clusters involved in DMOA-derived meroterpenoid biosynthesis. This *insA* gene cluster was heterologously expressed in *A. oryzae* NSAR1, a powerful platform for the refactoring of natural product biosynthesis in fungi and subsequently discovered several new meroterpenoids [26].

Unlike the approach used by Tang and Matsuda, our technology captures *unsequenced*, *undefined* BGCs as individual FACs for heterologous expression in a modified strain of the host *Aspergillus nidulans* (FAC-*An*HH) to yield a series of FAC-Trs. These FAC-Trs have been grown under a variety of culture conditions to investigate the production of new SMs. We report here ten unique FAC-Trs that yielded fourteen different natural products under identical fermentation conditions. Eleven of these compounds were not detected in FAC-*An*HH extracts. Of this discrete set, only the novel compound citreohybriddional (1) had been isolated previously from either PW2A or PW2B and only at low yield [27].

## 2. Results and discussion

### 2.1. FAC library construction, FAC-NGS and individual BGC capture as FACs

**Figure 1.**
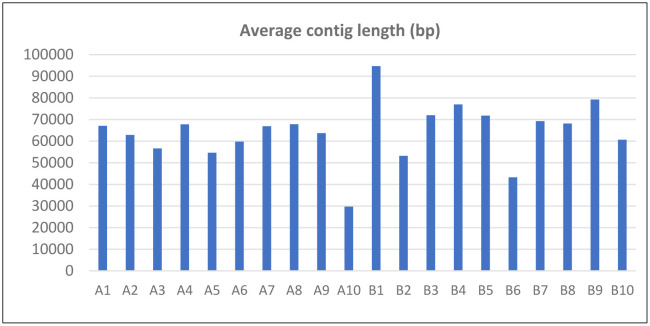
Average contig length of FAC pools. FAC pools: A1-A10 and B1-B10 are from *P. fuscum* (PW2A) and *P. camembertii/clavigerum* (PW2B), respectively.

Using FAC technology [24],[25] we successfully constructed unbiased “random shear” shuttle FAC libraries with average inserts of 120 kb (average 70 kb assembled contigs) from the unsequenced genomes of *P. fuscum* (PW2A) and *P. camembertii/clavigerum* (PW2B) (Figure 1).

We discovered 63 full-length BGCs from PW2A and 70 full-length BGCs from PW2B, respectively (Table 1). To date, we have generated 26 BGC-FACs, which were used to create FAC-Trs using FAC-*An*HH and a shuttle vector. The FAC libraries were sequenced, and priority was given to those that contained PKS gene clusters, because BPLA is produced through polyketide synthesis. Ten FAC-Trs were selected for this initial study.

**Table 1.** Predicted SM gene clusters from *P. fuscum* (PW2A) and *P. camembertii/clavigerum* (PW2B).

Predicted SM Gene Clusters	*PW2A*	*PW2B*
Polyketide synthases (PKS)	23	24
dimethylallyl tryptophan synthases (DMATS)	6	6
Nonribosomal peptide synthetases (NRPS)	17	17
Hybrid NRPS/PKS	11	3
Other	6	20
Total	63	70

Each of the ten FAC-Trs and the FAC-*An*HH were grown under identical culture conditions to those used in the axenic and co-culture experiments with PW2A and PW2B [23]. At time of harvest, each culture was extracted (unfiltered) with CHCl_3._ The crude CHCl_3_ extracts were analyzed by Liquid Chromatography/Mass Spectrometry (LC/MS) using Agilent Mass Hunter Work Station for data analysis and Nuclear Magnetic Resonance spectroscopy (NMR). Following the initial CHCl_3_ extraction, the aqueous filtrates were lyophilized, then extracted first with CHCl_3_-MeOH (1:1), and finally with MeOH, to access more polar SMs. The metabolites isolated from these more polar extracts will be described at a later date.

### 2.2. Secondary metabolites produced by FAC-transformants

**Figure 2.**
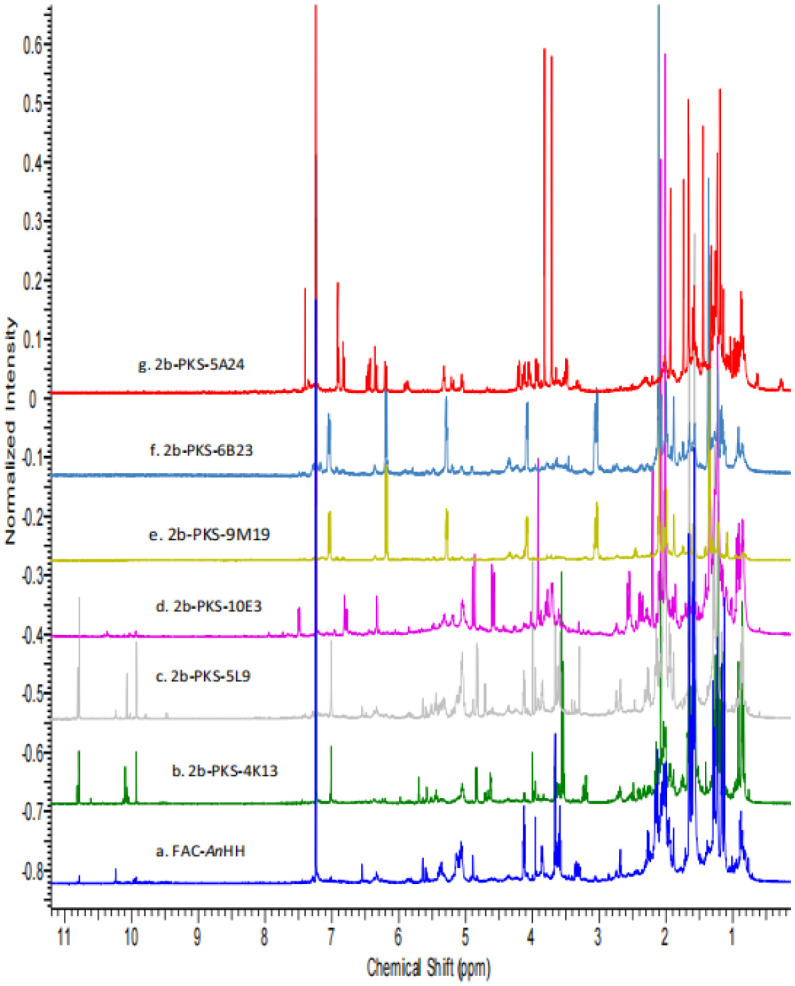
Stack plot comparing ^1^H NMR spectra of the crude CHCl_3_ extracts of FAC-*An*HH (a) and six FAC-Trs (b–g).

The identification of a SM usually requires isolation and purification using iterative chromatographic separations before it can be characterized using spectral methodology. However, if a compound is produced in sufficiently high yield, it can be readily discernable even in the crude extract. Although analysis of NMR spectra requires expertise, an untrained eye can recognize differences in the overall chemical shift patterns denoting unique compounds. The ^1^H NMR spectral data of six of the FAC-Trs (Figure 2 b–g) differed significantly from that of the FAC-*An*HH (Figure 2a). It was clear that there were compounds produced by specific FAC-Trs that were not apparent in the FAC-*An*HH extract. The spectra of 2bFACPKS-9M19 (2e) and 2bFACPKS-6B23 (2f) were virtually identical to each other even though they were derived from unrelated FAC-Trs. The spectrum of 2bFACPKS-5A24 (2g) differed significantly from the spectrum of any other FAC-Tr extract.

**Figure 3.**
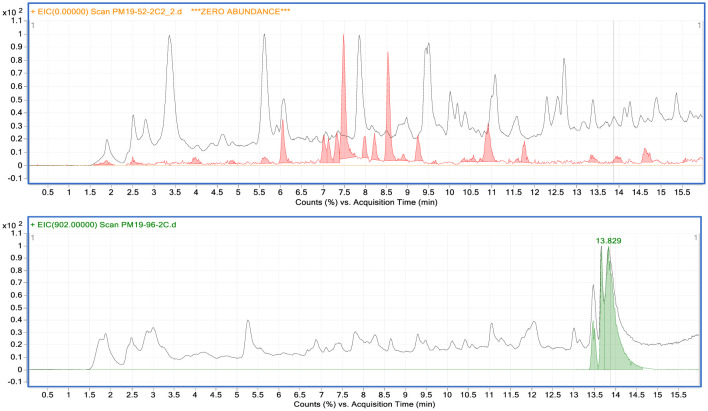
Comparison of LC/MS analyses (TICs) of the CHCl_3_ extracts of FAC-*An*HH (above) and FACPKS-5A24-3B, with UV spectra overlaid (250.4 nm) in red or green, respectively. The large peak at 13.6 minutes is in the FACPKS-5A24 extract is strobilurin G (2).

Each crude extract was also carefully analyzed by comparative LC/MS which provided evidence of additional compounds that were not apparent in the ^1^H NMR spectra. Mass spectrometry is a significantly more sensitive analytical tool than NMR, with theoretical instrument detection limits (IDL) as low as 10^-14^ g [28],[29]. Comparative LC/MS analyses of the CHCl_3_ extracts of the FAC-*An*HH and *FACPKS-5A24-3B* showing TICs (Total Ion Chromatograms) with UV traces overlaid are shown in Figure 3. The large peak at 13.6 minutes in the 5A24-3B extract is strobilurin G (2). There was no evidence of strobilurin production by the FAC-*An*HH, nor by any of the other FAC-Trs. LC/MS data of the crude CHCl_3_ extracts of all of the FAC-Trs and FAC-*An*HH are shown in Figure S1.

**Table 2.** Heterologous expression of BGC-FACS from Berkeley Pit isolates: PW2A and PW2B. Compounds identified by LC/MS analysis but not isolated and purified are denoted by *.

Gene Clusters	FAC-Transformants	Secondary Metabolites	% yield
A6-C18-S76	*An*-2aFACPKS-6B23-2B	asperlin (9)	61%
A6-C5-S7	*An*-2aFACPKS-7G5-2B	asperlin (9)	42%
B6-C12-S70	*An*-2bFACPKS-9M19-1	asperlin (9)	66%
B4-C7-S30-A	*An*-2bFACPKS-5A24-3B	strobilurin G (2)	35%
		strobilurin F (3)	17%
B8-C3-S6	*An*-2bFACPKS-10E3-2B	sequoiamonascin D (4)	3%
		sequoiatone A (5)	2%
		sequoiatone F (6)	2%
		penicillide (7)	4%
		dihydroxy-3,5,7-tri-methylisochroman (8)	24%
B9-C27-S74	*An*-2bFACPKS-1K15-2	asperugin A (11)	8%
		asperugin B (12)	6%
B1-C15-S42	*An*-2bFACPKS-1L15-1	asp A (11)* and B (12)*	
B4-C7-S30-B	*An*-2bFACPKS-4K13-2	citreohybbridional (1)	2%
		asperugin A (11)	7%
		asperugin B (12)	4%
B9-C27-S74	*An*-2bFACPKS-5L9-1	asperugin A (11)	1%
		asperugin B (12)	2%
B9-C27-S74	*An*-2bFACPKS-2J5-1	asp A (11)* and B (12)*	
	FAC-*An*HH	farnesol (10)	8%
		dihydroxy farnesol (13)	7%
		dihydroxy methyl farnesoate (14)	11%

**Figure 4.**
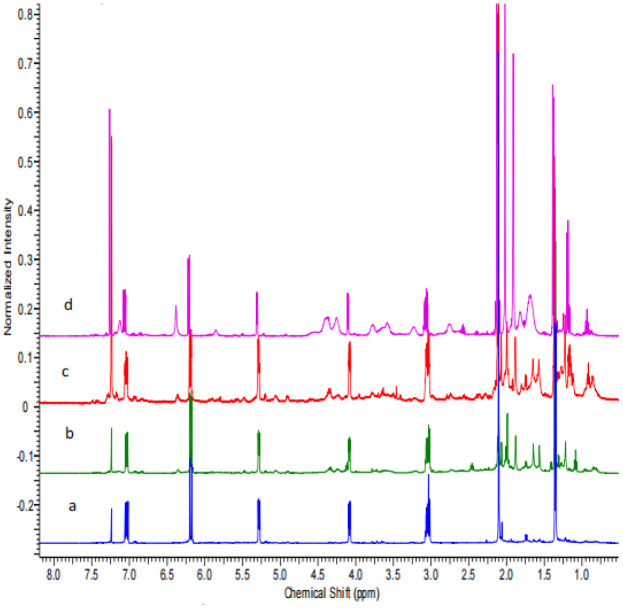
Comparison of the ^1^H NMR spectra of (a) pure asperlin (9) and the crude CHCl_3_ extracts of three independent FAC-Trs: (b) 2b-PKS-9M19-1; (c) 2a-PKS-6B23-2B; and (d) 2a-PKS-7G5-2B. Asperlin was not detected in the crude extract of the FAC-*An*HH. All spectra were run in CDCl_3_.

**Figure 5.**
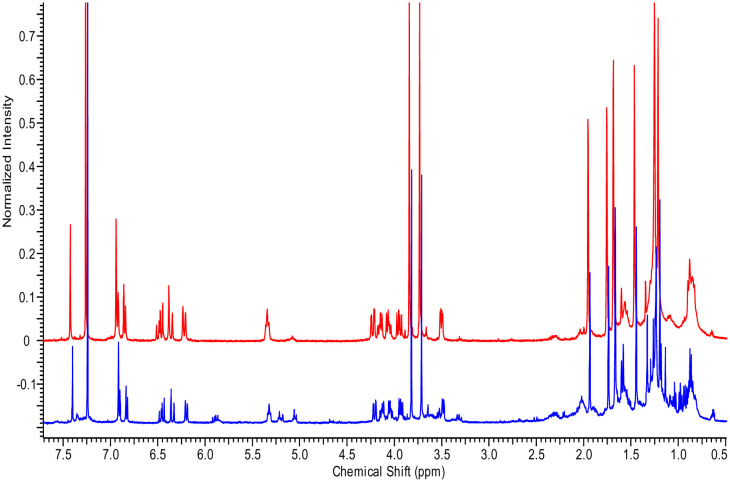
Comparison of the ^1^H NMR spectra of purified strobilurin G (red) and the crude CHCl_3_ extract (PM19-96B) of 2bFACPKS-5A24-3B (blue).

To date, we have identified fourteen SMs from ten FAC-Trs. The secondary metabolites reported here were purified using iterative HPLC, then characterized using 1D and 2D NMR spectroscopy and mass spectrometry. The masses of the purified samples were compared to the total mass of the crude organic extracts to determine % yield, and are the average of three replicated experiments (Table 2). Both asperlin (9) and strobilurin G (2) comprised >30% of the crude CHCl_3_ extracts of discrete FAC-Trs, unusually high % yields of single compounds from fungi grown in unoptimized conditions (Figures 4 and 5). Eleven of the compounds isolated in this study were unique to the FAC-Trs and undetectable in the FAC-*An*HH extract (Table 2; Table S1).

The ten BGC-FACs used to create the FAC-Trs were confirmed based on full-length re-sequencing by individual indexing Illumina sequencing. Two of these BGC-FACs were isolated from PW2A: *2aFACPKS-6B23-2B* and *2aFACPKS-7G5-2B*; and eight from PW2B: *2bFACPKS-1L15*, *2bFACPKS-2J5*, *2bFACPKS-4K13*, *2bFACPKS-5A24-3B*, *2bFACPKS-5L9*, *2bFACPKS-9M19*, *2bFACPKS-1K15-2* and *2bFACPKS-10E3-2B* (GenBank # MZ156759, MZ233785~MZ233793).

**Figure 6.**
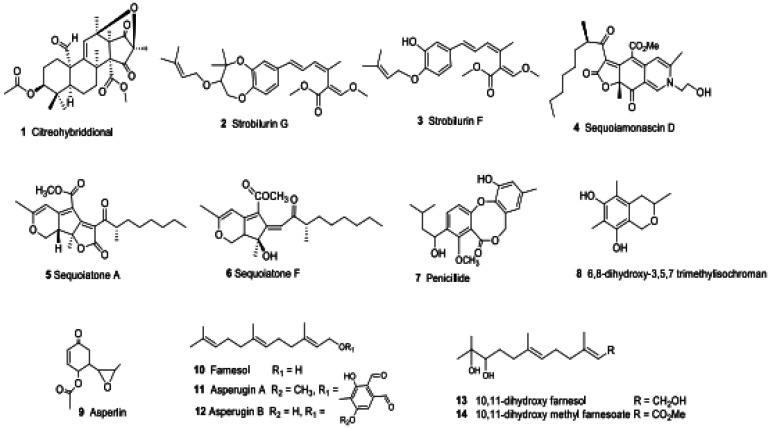
Secondary metabolites produced either by specific FAC-Trs through heterologous expression, or by the FAC-*An*HH, and subsequently isolated and elucidated.

The fourteen SMs (Figure 6) discovered in this study can be categorized as follows:

- Citreohybriddional (1) is a novel compound isolated exclusively from *2bFACPKS-4K13* and is undetectable in the FAC-*An*HH extracts. It is also the only compound isolated and characterized in this study that was previously isolated from co-culture of PW2A/PW2B and from *Penicillium turbatum* [27].
- Strobilurin F (3) and G (2), sequoiamonascin D (4), sequoiatone A (5), sequoiatone F (6), penicillide (7) and 6,8-dihydroxy-3,5,7-trimethylisochroman (8), asperlin (9), asperugin A (11) and B (12) were reported previously in the literature. In this study, these compounds were isolated from specific FAC-Trs, but were not detected in the axenic or co-culture extracts of PW2A or PW2B, or in extracts of FAC-*An*HH. The isolation of strobilurin F (3) and G (2) from a FAC-Tr was surprising, as this family of compounds has been previously isolated exclusively from the basidiomycetes *Strobiluris* and *Bolinea* [30]. This is the first report of their production by an ascomycete. Compounds 4–6 were previously isolated from a *Sequoia sempervirens* endosymbiont, *Aspergillus parasiticus* [31]–[33]. Penicillide (7) was previously isolated from a *Penicillium* sp [34]. 6,8-dihydroxy-3,5,7-trimethyl-isochroman (8) was previously reported exclusively as a SM of *Stachybotrys cylindrospora*, an endosymbiont of aspen trees [35]. It is interesting to note that although asperlin (9) has been isolated from several *Aspergillus* sp [36], and that asperugin A (11) and B (12) were previously isolated from *Aspergillus rugulosus* [37],[38], they were not detected in the crude extract of the *Aspergillus*-derived heterologous host, FAC-*An*HH.
- Farnesol (10), 10,11-dihydroxy farnesol (13) and 10,11-dihydroxy-methyl farnesoate (14) [39] were produced by both the FAC-*An*HH and discrete FAC-Trs. These compounds are potential precursors for the more complex asperugins, which were produced by several FAC-Trs but were not detected in the FAC-*An*HH extracts (Table 2, Table S1).

Once the structures of the SMs were determined, their data was compared to literature values for confirmation. The structure elucidations of 1 and 2, however, will be provided in more detail, as 1 is a new compound, and 2 is an unusual compound to be produced by an ascomycete FAC-Tr.

## 3. Characterization of secondary metabolites

### 3.1. Characterization of citreohybriddional (1) from FAC-Tr

**Figure 7.**
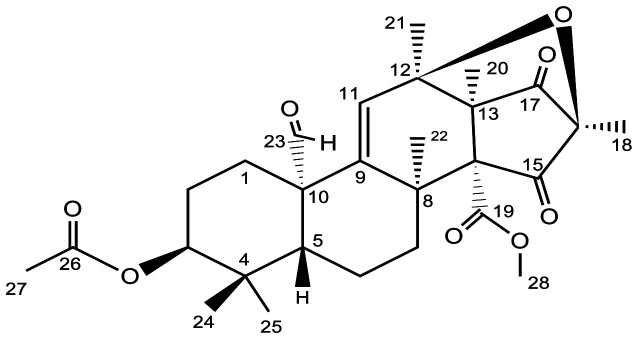
Structure of citreohybriddional (1) showing numbering scheme for correlation with NMR data.

Compound 1 (*2bFACPKS-4K13*) had a molecular formula of C_28_H_36_O_8_ established by HREIMS ([M+23]^+^ = 523 amu), with 11 degrees of unsaturation. NMR data (C_6_D_6_) (Table 3) confirmed the presence of two ketone carbons (δ_C_ 202.9, 202.6), one aldehyde (δ_C_ 201.2, δ_H_ 9.80, s), two ester carbons (δ_C_ 170.0, 168.3) and one trisubstituted double bond (δ_C_ 148.4, 126.8; δ_H_ 5.53, s). The five carbonyl carbons and one olefin accommodated 6 degrees of unsaturation, so compound 1 was pentacyclic. ^1^H NMR data confirmed 8 methyl singlets, including a methyl ester (δ_H_ 3.00), and an acetate methyl (δ_H_ 1.53). The large number of methyl groups relative to the total number of carbons suggested a terpenoid component to the skeleton. Careful examination of the data indicated that this compound was identical to citreohybriddional (1), (Figures 6 and 7) which we had isolated from PW2A-PW2B co-culture and from *P. turbatum* [27]. (NMR data set included as Figures S2 a–e.)

PW2A and PW2B have been grown multiple times (homologous expression) both as axenic cultures and in co-culture, under identical conditions to those used with the FAC-Trs. Of all of the SMs identified in this study, only citreohybriddional (1) was produced in a PW2A/PW2B co-culture experiment. This was verified by comparison of the NMR and LC/MS data of pure and co-culture extracts of these two fungi with that of all of the FAC-Tr extracts.

**Table 3.** ^1^H (500 MHz) and ^13^C (125 MHz) NMR data for citreohybriddional (1) in C_6_D_6._

	Δ_13_	δ_H,_ mult (*J =* Hz)	HMBC	NOESY
1	27.4, CH_2_	α 2.21, dt (*J* = 12.2, 3.2) β 1.47, m		H-11 H-11
2	24.3, CH_2_	β 1.65, m, α 1.55, m	H-1β	H-25
3	76.9, CH	4.73, bt (*J* = 2.7)	H-24, H-25	H-2α, H-2β, H-24, H-25
4	37.8, C		H-24, H-25	
5	46.8, CH	1.90, m	H-24, H-25	
6	17.0, CH_2_	α 1.90, m β 1.49, m	H-5, H-7α	
7	33.6, CH_2_	α 3.11, m β 2.08, dm (*J* = 13.6)	H-22	H-6α, H-6β
8	40.4, C		H-11, H-22	
9	148.4, C		H-22	
10	55.6, C		H-11, H-23	
11	126.8, CH	5.53, s	H-21	H-1α, H-1β, H-21
12	76.5, C		H-11, H-20, H-21	
13	53.6, C		H-11, H-20, H-21	
14	72.4, C		H-20, H-22	
15	202.6, C		H-18	
16	75.5, C		H-18	
17	202.9, C		H-18, H-20	
18	8.3, CH_3_	1.36, s		
19	168.3, C		H-28	
20	10.8, CH_3_	1.14, s		H-21, H-22
21	24.5, CH_3_	0.99, s	H-11	H-20
22	26.8, CH_3_	1.39, s		H-20, H-23
23	201.2, CH	9.80, s	H-5	H-22, H-25
24	27.2, CH_3_	0.80, s	H-3, H-25	H-3
25	21.9, CH_3_	0.65, s	H-24	H-3, H-23
26	170.0, C		H-27	
27	20.8, CH_3_	1.53, s		H-3
28	52.0, CH_3_	3.00, s		

All assignments are based on COSY, HSQC and HMBC experiments, *J* is in Hz.

We propose that with the exception of the citreohybriddional BGC, the BGCs of the compounds isolated from these FAC-Trs are silent in the original host fungi. We recently published the isolation and characterization of 1 from *P. turbatum*, and the data from the compound isolated from *2bFACPKS-4K13* is identical [27].

### 3.2. Characterization of Strobilurin G (2) from FAC-Tr

**Figure 8.**
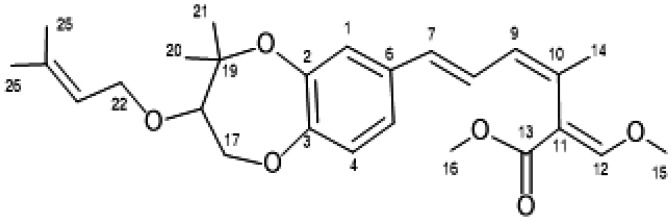
Numbering scheme for Strobilurin G (**2**) established by Fredenhagen [40].

If the production of a relatively new compound from a FAC-Tr associated with PW2B was exciting, then the isolation and characterization of the strobilurins from *2bFACPKS-5A24-3B* was surprising. Strobilurin G (2) (Figures 6 and 8) had a molecular formula of C_26_H_34_O_6_ established by HREIMS ([M+18]^+^ = 460 amu), with 10 degrees of unsaturation. The ^1^H NMR (Table 4 and Table S2) provided evidence of seven allylic/aromatic protons. Two protons (δ_H_ 6.85 and 6.92) had coupling constants of 8.1 Hz, typical of ortho-coupled aromatic protons, so we proposed an aromatic ring system. The large coupling constants 10.6 Hz and 15.6 Hz associated with three protons (δ_H_ 6.20, 6.37 and 6.46) were typical of two conjugated double bonds. ^1^H NMR data also indicated three allylic methyls (δ_H_ 1.67, 1.74 and 1.94); two methyls deshielded by proximity to a carbonyl group (δ_H_ 3.82 and 3.72): and two aliphatic methyl groups.

^13^C NMR data provided additional information. There were 15 sp^2^ hybridized carbons, which accommodated six aromatic carbons, eight olefinic carbons and a carbonyl carbon, providing nine degrees of unsaturation. This required an additional ring to accommodate ten degrees of unsaturation. Two of the aromatic carbons were deshielded (δc 146.7 and 150.0), indicating attachment to oxygens. These initial observations were corroborated with input from 2-dimensional (2D) correlation spectroscopy, which facilitated assignments of proton-proton coupling, and both short range and long-range proton-carbon coupling. A more detailed structure elucidation as well as 2D-NMR data are provided in Figures S3a-e. [Figure S4 provides a graphical depiction of proton-proton and proton-carbon connectivity derived from NMR data of strobilurin G (2). Figure S5 provides ^1^H NMR of strobilurin F (3).]

Following elucidation of (2), we compared the NMR and mass spectral data of our compound with the literature values of strobilurin G isolated from *Bolinea lutea* [40], and produced by total synthesis [41] (Tables 4 and 5). The data were consistent. A reviewer suggested that our cultures had been contaminated by strobilurin G (2) from an exogenous source, but our lab has never worked with either basidiomycetes in general or strobilurin in particular, so that possibility is highly unlikely.

**Table 4.** Comparison of ^1^H NMR data of strobilurin G (2) isolated from *Bolinia lutea*, produced by total synthesis and isolated from the FAC-Tr *2bFACPKS-5A24-3B* (Figure S3a). For all three data sets, 2 was dissolved in CDCl_3_.

Proton	Strobilurin G (*Bolinia lutea*)		Strobilurin G (Synthesis)		Strobilurin G (Fac-tr.)	
1	6.94	br s	6.93	d, *J* = 2	6.93	br s
4	6.85	dd, *J* = 8.5	6.85	d, *J* = 1, 7.9	6.85	dd, *J* = 1.14, 8.1
5	6.93	dd, *J* = 8.5	6.92	dd, 2.1, 7.9	6.92	dd, *J* = 1.14, 8.1
7	6.37	d, *J* = 15.5	6.37	d, *J*= 15.6	6.37	d, *J* = 15.6
8	6.48	dd, *J* = 10.5, 15.5	6.48	dd, *J* = 10.7, 15.6	6.46	dd, *J* = 10.6, 15.6
9	6.22	qd, *J* = 10.5	6.22	dd, *J* = 1, 10.6	6.20	dd, *J* = 1.0, 10.6
12	7.43	s	7.42	s	7.42	s
14	1.96	br s (3H)	1.96	br s (3H)	1.94	s, (3H)
15	3.84	s (3H)	3.84	s (3H)	3.82	s (3H)
16	3.73	s (3H)	3.73	s (3H)	3.72	s (3H)
17(a)	4.23	dd, *J* = 3, 12.5	4.23	dd, *J* = 3.2, 12.4	4.23	dd, *J* = 3.1, 12.4
17(b)	3.95	dd, *J* = 8, 12.5	3.95	dd, *J* = 7.9, 12.3	3.93	dd, *J* = 7.9, 12.4
18	3.50	dd, *J* = 3, 8	3.49	dd, *J* = 3.2, 7.9	3.48	dd, *J* = 3.1, 7.9
20	1.21	s	1.21	s	1.19	s
21	1.47	s	1.47	s	1.45	s
22a	4.15	br dd, *J* = 6.5, 11.5	4.15	dd, *J* = 6.8, 11.3	4.13	dd, *J* = 6.8, 11.6
22b	4.06	br dd, *J* = 7, 11.5	4.06	dd, *J* = 6.8, 11.3	4.04	dd, *J* = 6.8, 11.6
23	5.34	t, *J* = 1.5, 6.5, 7	5.34	tqq, *J* = 6.8, 1.5, 1.5	5.32	tqq, *J* = 6.8, 1.5, 1.5
25	1.76	br s (3H)	1.76	br s (3H)	1.74	d (3H), *J* = 1.5
26	1.69	br s (3H)	1.69	br s (3H)	1.67	d (3H), *J* = 1.5

**Table 5.** Comparison of ^13^C NMR data of strobilurin G (**2**) isolated from the basidiomycete *Bolinia lutea*, produced by total synthesis and isolated from FAC-Tr *2bFACPKS-5A24-3B*. All three compounds were dissolved in CDCl_3_.

Carbon#	Strobilurin G *Bolinea lutea*	Strobilurin G Synthetic	Strobilurin G Fac-Tr
1	121.6	121.7	121.5
2	146.8	146.9	146.7
3	150.8	150.9	150.0
4	120.6	120.7	120.4
5	122.4	122.5	122.3
6	133.7	133.8	133.6
7	130.4	130.5	130.4
8	125.7	125.8	125.5
9	129.8	129.9	129.7
10	130.8	130.9	130.6
11	110.8	110.9	110.7
12	158.9	159.0	158.8
13	167.9	167.9	167.7
14	23.7	23.8	23.5
15	61.9	62.0	61.7
16	51.6	51.7	51.4
17	68.7	68.8	68.6
18	81.9	82.1	81.8
19	80.6	80.7	80.6
20	27.7	27.8	27.5
21	20.8	20.9	20.6
22	67.3	67.5	67.2
23	120.9	121.0	120.8
24	137.5	137.6	137.4
25	25.8	25.9	25.7
26	18.1	18.2	17.9

This was the most unusual finding from our set of FAC-Trs. AntiSMASH was used to characterize the captured BGC from *2bFACPKS-5A24-3B*. There were no homologous protein sequences between our FAC-BGC and the strobilurin BGC isolated from the basidiomycete *Strobilurus* [42]. We are currently evaluating our BGC and the comparison of the ascomycete biosynthesis of the strobilurins to that of the previously defined basidiomycete biosynthesis.

## 4. Comparison of FAC-BGCs with published BGCs in the Genbank database

The genomes of PW2A and PW2B showed little similarity to each other (GenBank # MZ156759, MZ233785~MZ233793). In this study, the 2aFACs of the PW2A genome were very diverse, while the eight 2bFACs from PW2B aligned with genomic regions of *P. expansum*, with >95% ~100% identity overlap. Both PW2B and *P. expansum* produce patulin and citrinin in culture (23, 25). The complicated biosynthetic pathway of patulin has been confirmed, but only two genes encoding 6-methylsalicylic acid synthase and isoepoxydone dehydrogenase have been identified in *P. expansum* [41]. However, neither citrinin nor patulin were produced by this initial set of FAC-Trs.

BLAST (Basic Local Alignment Search Tool) technology was used to identify homologies between our FAC-BGC sequences and fungal sequences in the Genbank database. For PW2A sequences, we found that *2aFACPKS-7G5* does not have sequence homology >1.8kb to any other fungal genome in the database. However, the asperlin (9) producer *2aFACPKS-6B23* has large genomic regions (20~50 kb) that are homologous to chromosomal regions of *Aspergillus sojae*, *Aspergillus flavus* and *Coccidioides posadasii/immitis*.

FAC-Tr *2bFACPKS-10E3-2B* contains a single PKS-BGC but produced six different compounds: sequoiamonascin D (4), sequoiatone A (5), sequoiatone F (6), penicillide (7), 6,6-dihydroxy-3,5,7-trimethylisochroman (8) and asperugin A (11). This PKS gene has 29% homologous protein sequence identity to the *2362MpPKS5* gene of the *Monascus pilosus* azaphilone pigment BGC cluster, which is associated with the synthesis of azaphilone type pigments [43],[44]. Azaphilone pigments include rubropunctatin, which is related to compounds 4–6. Sequoiamonascin D (4) and sequoiatones A and B were originally isolated from the endophyte *Aspergillus parasiticus*, which was harvested from the bark of *Sequoia sempervirens* [31]–[33]. (Figure S6).

*2bFACPKS-10E3-2B* also shares 30% identity with the *mpdG* (AN0150) gene of the *A. nidulans* monodictyphenone (*mdp*) BGC [45] and 31% identity with the *ptaA* gene of the diphenyl ether BGC for pestheic acid (pta) biosynthesis in the plant endophyte *Pestalotiopsis fici*, two compounds that are closely related to penicillide (**7**) [46], (Figure S7). There is also a gene with 22% identity to the amino oxidase/esterase-2367B gene of the *M. pilosus* azaphilone pigment BGC [45]. A transcription factor gene flanking this BGC has homologous protein sequences to *mdpE* (AN0148) and *ptaR2*, at 34% and 36% identities, respectively [45],[46].

FAC clone *2bFACPKS-5A24-3B* yielded strobilurins F (3) and G (2), two potent fungicides that have previously been isolated exclusively from the basidiomycetes *Strobilurus tenacellus*, *Cyphellopsis anomala* and *Bolinia lutea* [47]. This was an unexpected discovery, as there is little overlap in the SMs isolated from ascomycetes and basidiomycetes, and their respective biosynthetic pathways may differ, sometimes significantly [47],[48]. This was the most unusual finding from our set of FAC-Trs to date. AntiSMASH was used to characterize the captured BGC from *2bFACPKS-5A24-3B*. There were no homologous protein sequences between our FAC-containing BGC and the strobilurin BGC isolated from *Strobilurus* [42]. We are currently evaluating our BGC and the comparison of the ascomycete biosynthesis of the strobilurins to that of the previously defined basidiomycetes. Our FAC-BGC shares sequence homology with 44%–65% identities to all seven genes of the entire phomasetin (*phm*) BGC of *Pyrenochaetopsis sp*. *RK10-F058* [49] (Figure S8C). Although FAC clones *2bFACPKS-5A24-3B* and *2bFACPKS-4K13* were identified by the same pair of PCR primers, they contain totally different sequences and BGCs by re-sequencing; therefore, the predicted BGCs were re-named as *B4-C7-S30-A & B* (Table 2).

*2aFACPKS-6B23-2B*, *2aFACPKS-7G5-2B* and *2bFACPKS-9M19* shared no homologous BGCs with each other, yet they all produced asperlin (9) at > 40% yield. Of the three asperlin producing clones, only *2aFACPKS-6B23-2B* has homologous protein sequences (20%–48% identities) of seven genes of the 10-member silent asperlin (*aln*) BGC in *Aspergillus nidulans* [50] (Table S1 and Figure S8A and B). We propose that both *2aFACPKS-7G5-2B* and *2bFACPKS-9M19* are actually activating the silent *aln* BGC in the FAC-*An*HH heterologous host.

Two sequence-confirmed FACs, *2bFACPKS-2J5 and 2bFACPKS-5L9*, produced asperugin A (11) and B (12) in culture. Although these two FAC-Trs were identified by the same PCR primers, both had completely different DNA sequences and no significant homologous gene(s). *2bFACPKS-1L15* and *2bFACPKS-1K15*, also produced 11 and 12 in culture but have not yet been sequence-confirmed. These FAC-Trs represent at least four different predicted BGCs, but all produced 11 and12. Therefore, we propose that the asperugins were actually produced by silent genes associated with the FAC-*An*HH, perhaps as an aspernidine A intermediate [51]. The asperugins were not detected in the extracts of the FAC-*An*HH.

*2bFACPKS-2J5* BGC is homologous to the entire citrinin (*cit*) BGC from *Monascus ruber* isolate M7. The gene order is also conserved among these homologous BGCs of *Monascus ruber* isolate M7, *P. camembertii/clavigerum* (PW2B) and its closely related fungus, *P. expansum* [52] (Table S1 and Figure S8D).

FAC-Tr *2bFACPKS-4K13* produced citreohybriddional (1). The citreohybridones and related meroterpenoids, as well as their BGCs, have been extensively studied. We have updated the homologous BGC alignment based on the published data (Table S1 and Figure S9).

Although compounds 2–14 have been previously reported in the literature from diverse fungi, citreohybriddional (1) has been only recently reported by the Stierle lab and is novel. We have determined the absolute structure of 1, which is published elsewhere [27].

Following the isolation and characterization of compounds 1–14 from the FAC-Trs, we compared their LC/MS data with that of the axenic and co-culture experiments of PW2A/PW2B. Of all of the compounds isolated from the various FAC-Trs, only evidence of citreohybriddional (1) could be found. It was produced in very low yield, exclusively in a co-culture fermentation of PW2A and PW2B.

## 5. Conclusion

We have shown that FACs facilitate the activation of cryptic or silent fungal BGCs in FAC-Trs, which may result in high yields of specific secondary metabolites under standard fermentation conditions, (Table 2; Table S1).

In 2015 we performed similar transformations using BGCs from three *Aspergillus* species (*A. aculeatus, A. terreus* and *A. wentii*) with the same FAC-*A. nidulans* heterologous host [24]. We have demonstrated that our approach is equally effective with *Penicillium* species. We proposed that the *Penicillium* BGCs are recognized by the *Aspergillus* transcription machinery because the two genera are closely related. These initial studies suggest that this is indeed the case. We are not sure why are these *Penicillium* BGCs are switched on in the *Aspergillus* host but not in their own host organism. We are currently exploring this phenomenon to understand the underlying mechanisms associated.

Sequencing data confirmed that PW2A and PW2B have minimal DNA sequence homology. We directly captured intact SM-BGCs by NGS, facilitated FAC heterologous expression with high compound production, enabled direct analysis of the crude extracts of the FAC-Trs by NMR and LC/MS and determined the structures of fourteen natural products produced by FAC-Trs specifically. Fully understanding the mechanisms involved in this powerful technology, and further scale-up of the process, could greatly enhance fungal NP discovery pipelines.

## 6. Material and methods

### 6.1. Construction of FAC library and FAC-NGS analysis

Using Fungal Artificial Chromosome (FAC) technology [24],[25] we have successfully constructed unbiased “random shear” shuttle FAC libraries with average inserts of 100–150 kb (Figure S10) from the unsequenced genomes of *P. fuscum* (PW2A) and *P. camembertii*/*clavigerum* (PW2B). We performed 2 Illumina Miseq runs with v3 chemistry (2 x 300 bp) and generated ~32Gb of sequencing data. By setting up an auto-assembling, annotation and antiSMASH pipeline for the sequencing data analysis, we assembled the FAC pools as individual FACs (~1,000 contigs, >100kb each and ~2,500 contigs, >50 kb each.). We discovered at least 63 full-length BGCs from PW2A and 70 full-length BGCS from PW2B, respectively. Of 133 predicted BGCs, we transformed 26 BGC-FACs using the heterologous fungal host, *A. nidulans* (FAC-*An*HH). The protocol for the creation of unbiased FAC libraries that averaged at least 100 kb insert-size of *Penicillium fuscum* and *P. camembertii/clavigerum* has been described [54]. Each FAC library encompassed 20 plates (384-well), or 7,680 FAC clones in total, which equaled at >15 x genome coverage of a 50 Mb fungal genome (7,680 x 100 kb/50 Mb x 1,000). In the first pass, we prepared the pooled FAC DNAs from each of ten 384-well plates per FAC library, an Illumina true-seq library for each FAC pool with an index, total 20 indexing Illumina true-seq libraries from the above 2 FAC libraries.

### 6.2. Extraction of Fungal DNA

Fungal genomic DNA was extracted from lyophilized mycelia of *P. fuscum* (PW2A) and *P. camembertii/clavigerum* (PW2B) using a modified method [54]. Briefly, 20 grams of fungal mycelia was frozen in liquid nitrogen, stored at −80 °C, then ground into a fine powder. The powder was resuspended in 20 mL of LETS buffer, mixed by inverting the tube several times, then diluted with 20 ml of phenol:CHCl_3_:isoamyl alcohol. After gentle mixing, samples were spun for 10 minutes (4 °C, 4,000 rpm). The supernatant was transferred to a fresh tube and an equal volume of phenol:CHCl_3_:isoamyl alcohol was added and the sample was spun as previously described. The supernatant was transferred to a new tube to with an equal volume of isopropanol. High molecular weight (HMW) threads of genomic DNA formed, which were washed with ethanol, dried, then dissolved in Tris-EDTA buffer (TE). The fungal HMW genomic DNA were > 50kb in size. (Further details are provided in the *Supplemental appendix*).

### 6.3. DNA ligation

The above fungal HMW DNA was mixed with 500 µL of 1% low-melting temperature agarose in miniQ water, end-repaired with the HMW gDNA repairing kit (intactgenomics.com) in a total volume of 2,000 µL with 40 µL of the end repairing enzymes which were heat inactivated (70 °C, 15 min). The resulting DNA was ligated with BstXI adaptors (40 µL of 100 µM each) in a total volume of 2,800 µL consisting of a ligation reaction of 40 µL of ligase (2 U/µL, Intact Genomics). Eight fractions of gel-fractionated DNA fragments ranging from 100 to 200 kb were purified by PFGE. Purified large DNA fragments (about 50 µL 1–3 ng/µL each fractions) were ligated into the cloning-ready pFAC BstXI shuttle vector at 16 °C for ~18 hours. Next, the ligated DNA mixture was electroporated into competent *E. coli* cells (BAC/FAC *E. coli* 10B Replicator cells, Intact Genomics). Small-scale ligations and transformations (1 µL DNA per 20 µL cells) were used to judge the cloning efficiency. The insert sizes of 8 x 45 random FAC clones were determined and confirmed to include inserts of about 100 kb (Figure S1a/b). Once the suitability of the ligated DNA was confirmed, large-scale ligations and transformations were conducted to achieve at least 7,680 clones for colony picking (20 x 384-well plates) for the unbiased shuttle FAC libraries.

### 6.4. FAC Pooling and Illumina-index-sequencing of FAC pools

Individual FACs were grown in 15 mL tubes. For FAC libraries, each FAC clone of the first 10 plates of FAC libraries of PW2A and PW2B were duplicated in a 384-deep-well plate with Terrific Broth (TB) Medium. TB medium: Yeast extract, 24 g, tryptone, 20 g, dissolved in 900 mL; Phosphate buffer: 0.17 M KH_2_PO_4_ and 0.72 M K_2_HPO_4_ in 100 mL. The solutions were autoclaved separately, then mixed together after cooling to room temperature. Finally, 8 mL of filter-sterilized 50% Glycerol, Chloramphenicol to 12.5 ug/mL and arabinose to 0.01% was added. The duplicated FAC plates were grown in shaking incubator at 37 °C, 200 rpm for 24 h. Individual FAC-DNAs or FAC-DNA pools from individually grown FAC cells were pooled together using a common alkali-plasmid/BAC DNA isolation method. Each FAC-DNA or pool was dissolved in 300 uL of 10 mM TrisHCl (pH 8.0). Twenty FAC plate-pools (A1-A10, B1-B10) and 40 sub-pools (16 row pools and 24 column pools) were created for each 384-well FAC plate, besides individual FACs. Further details are provided in the *Supplemental appendix*.

### 6.5. Selecting and confirming 26 BGC-FACs

Based on the predicted BGC-FAC sequences, we designed primers of each key gene and flanking genes at the predicted BGC boundary of the predicted BGC. We then used PCR to screen and confirm the results against the FAC pools and candidate BGC-FACs.

### 6.6. FAC heterologous expression in A. nidulans

A modified PEG-calcium based transformation method [22],[53],[54] was adapted to improve and simplify the transformation of 100 kb BAC-FACs. Briefly, we simplified the *A. nidulans* protoplast preparation by fixing the time in each step without the protoplast purification step to obtain the crude protoplast/cell mixture at concentration of about 3 x 10^7^.

For BGC-FAC-transformation: In a 1.7 mL Eppendorf tube, 10 µL (2 µg) of BGC-FAC DNA was added to 100 µL of STC (1.2 M Sorbitol, 10 mM Tris–HCl, 10 mM CaCl_2_ pH 7.5). To this solution, 100 µL containing 3 x 10^7^ *A. nidulans* RJW256 protoplasts/cells was added with gentle mixing, then placed on ice for 50 min. 1.25 mL of 30% PEG 4,000 with 50 mM CaCl_2_ were added and the solution was mixed gently, then incubated for 10 min at room temperature. The entire transformation mixture was plated onto regeneration media plates (GMM with 1.2 M of sorbitol, 1 mL of 0.1% Pyridoxine/L,15 g of agar/L) to obtain BGC-FAC-Trs.

### 6.7. Culture conditions for creation of FAC-Trs and FAC-AnHH

At Intact Genomics, independent *A. nidulans* transformants of BGC-FACs were selected and re-grown on GMM plates with 1 mL of 0.1% Pyridoxine/L, 15 g of agar/L, without sorbitol. Three independent *A. nidulans* BGC-FAC strains with different morphotypes (if present) were selected for each of 26 BGC-FACs fermentation experiments. FAC-*An*HH was grown simultaneously under identical conditions. These [3 x 26] FAC-Trs were sent to the Stierle lab for fermentation and discovery of natural product production.

### 6.8. Culture conditions for heterologous expression of FAC-BGCs and FAC-AnHH

In the Stierle lab, PDB-pyr broth (4.0 g potato starch/L and 20 g dextrose/L, with 1 mL of 0.1% pyridoxine/400 mL H_2_O) was used for fermentation experiments. Ten of the 26 FAC-Trs sent from Intact Genomics were randomly chosen for the initial study, as reported here.

An agar cube (8 mm^3^) impregnated with selected FAC-Tr mycelium was added to each culture flask containing 400 mL of PDB-pyr. The cultures were in triplicate grown for 9 days, shaken at 190 rpm, 25 °C. These same conditions were also used to grow PW2A and PW2B (the source organisms) to facilitate comparison between SMs of FAC-Trs and their source organisms. At time of harvest, each organism was thoroughly extracted with CHCl_3_. Each extract was analyzed by ^1^H NMR spectroscopy and under identical conditions. The filtrate (aqueous portion) was lyophilized and further extracted with CHCl3-MeOH (1:1) and MeOH and analyzed by NMR and LC/MS. However, only the CHCl_3_ extract will be discussed in this report.

FAC-Tr extracts were then carefully analyzed by LC/MS (conditions below). Using Agilent Mass Hunter Qualitative Analysis, we could directly compare each CHCl_3_ extract with that of the FAC-*An*HH using Total Ion Chromatogram (TIC), Diode Array Detection (200 nm–800 nm absorption) for UV analysis, Base Peak (BP), or specific Extracted Ion Chromatogram (EIC) detection. EIC is created by plotting the intensity of the signal observed at a chosen mass-to-charge value as a function of retention time. This allows direct comparison of LCs run under identical conditions. which was useful in determining which compounds were unique to specific FAC-Trs and which were present in several transformants and/or the FAC-*An*HH as well.

### 6.9. Analysis of crude fungal extracts with NMR spectroscopy and LC/MS

Nuclear magnetic resonance (NMR) spectra (1D and 2D) were obtained using FAC-Tr crude extracts with a Varian 500 MHz, or a Bruker Avance 400 MHz spectrometer. Chemical shift values (δ) were given in parts per million (ppm), and coupling constants (*J*) were in Hz. Chemical shifts were recorded with respect to the deuterated solvent shift (CDCl_3_: δ_H_ 7.24 for ^1^H NMR and δ_C_ 77.0 for ^13^C NMR; C_6_D_6_: δH 7.16 for ^1^H NMR and δ_C_ 128.4 for ^13^C NMR; MeOH-D_4_: δ_H_ 3.30 for ^1^H NMR and δ_C_ 49.0 for ^13^C NMR; ). Liquid Chromatography/Mass Spectrometry (LC/MS) experiments were run on Agilent 6520 Q-TOF-LC/MS using a Phenomenex Gemini NX-C18 column. The LC was run in reverse phase gradient mode from 50% CH_3_CN/H_2_O with 0.1% formic acid to 100% CH_3_CN over 15 minutes, then held at 100% CH_3_CN for 4 minutes. All solvents used were spectral grade or distilled prior to use.

### 6.10. Isolation and identification of secondary metabolites in FAC-vector and FAC-transformants

After each extract was thoroughly analyzed by NMR and LC/MS, individual compounds were purified using iterative flash silica gel chromatography followed by HPLC. Each CHCl_3_ extract was fractionated by flash silica gel column chromatography using IPA-hexanes in a stepwise gradient system of increasing polarity, starting with 5% IPA to 100% IPA (10%, 20%, 50% IPA), followed by 100% MeOH. Each fraction was then analyzed by NMR and LC/MS. Fractions that contained compounds of interest were further resolved using semi-preparative silica gel HPLC [Varian Dynamax Microsorb 100-5] in gradient mode from 5% IPA-hexanes to 100% IPA over 60 min. The structures of pure compounds were determined using 1D-NMR and 2D-NMR techniques. The identity of each compound was confirmed by comparison to published spectra.
