# Supplementary material for: Activation of cryptic biosynthetic gene clusters by fungal artificial chromosomes to produce novel secondary metabolites
Source: AIMS Microbiol. 2023 Dec 18;9(4):757–79. doi: 10.3934/microbiol.2023039 (PMC10758572; doi:10.3934/microbiol.2023039)
Supplement: Supplementary file 1 [file microbiol-09-04-039-s001.pdf]

AIMS Microbiology

AIMS Microbiology, 9(4): 757–779.
DOI: 10.3934/microbiol.2023039
Received: 25 August 2023
Revised: 07 December 2023
Accepted: 08 December 2023
Published: 18 December 2023

http://www.aimspress.com/journal/microbiology

*Research article*

# Activation of cryptic biosynthetic gene clusters by fungal artificial chromosomes to produce novel secondary metabolites

**Chengcang C. Wu[1]*†, Andrea A. Stierle[2]*†, Donald B. Stierle[2]†, Hongyu Chen[1], Michael Swyers[1], Timothy Decker[1], Emili Borkowski[1], Peter Korajczyk[1], Rosa Ye[1] and Niel Mondava[2]**

[1] Intact Genomics, Inc. 1100 Corporate Square Drive, Suite 257, St Louis, Missouri, 63132, USA
[2] Department of Biomedical and Pharmaceutical Sciences, University of Montana, Missoula, Montana 59812, USA

* **Correspondence:** Email: cwu@intactgenomics.com; andrea.stierle@mso.umt.edu

†These authors contributed equally to this work.

## 1. Materials and methods

### *1.1. Extraction of fungal DNA*

Fungal genomic DNA was extracted from lyophilized mycelia of *P. fuscum* (PW2A) and *P. camemberti* (PW2B) using a modified method. Briefly 20 grams of fungal mycelia was frozen in liquid nitrogen, stored in 80 °C, or shipped with dry ice between our labs, broken up lyophilized hyphae into a fine powder by grinding. The fungal mycelia powder was resuspended in 20 mL of LETS buffer: 0.1M LiCl, 20 mM EDTA (pH8), 10mM Tris-HCl (pH8), and 0.5% SDS. Mix by inverting the tubes several times and leave the samples on bench for 5min. Add 20 ml of Phenol:$CHCl_3$:isoamyl alcohol (25:24:1). Mix by gentle inverting 10–15 times. Let samples on bench for 5 min. Spin 10 minutes at 4 °C at 4,000 rpm, transfer supernatant to a new tube, and add equal volume of Phenol:$CHCl_3$:isoamyl alcohol and repeat the previous steps, transfer supernatant to a new tube, add equal volume of isopropanol, mix well, hook up the cotton thread-like high molecular weight (HMW) genomic DNA, wash with 70% ethanol, dry for 3 min, and gently dissolve the fungal HMW genomic DNA in 500 µL of TE. The

fungal HMW genomic DNA should be >50kb ~megabases in sizes.

*1.2. Construction of FAC library and FAC-NGS analysis*

The *Escherichia coli* (*E. coli*) strain used is Intact Genomics BAC/FAC E. coli 10B Replicator with the genotype of F-mcrA Δ(mrr-hsdRMS-mcrBC) endA1 recA1 Φ80dlacZΔM15 ΔlacX74 araD139 Δ(ara,leu)7697 galUgalK rpsL nupG (attL araC-PBAD-trfA250 bla attR) λ-. The FAC-vector is the portion sequence labeled as "pFAC cloning vector", 16,040 bp of the shuttle vector AtFAC9J20, complete sequence (GenBank #: KX449366.1). The fungal heterologous host, *Aspergillus nidulans* (*A. nidulans*) strain RJW256 (pyrG89, pyroA4, Δnku70::argB, ΔST::afpyrG, veA1) was obtained by a sexual cross between LO4641 (riboB2, pyroA4, ΔST::AfpyrG, ΔAN7909::afpyrG, Δnku70::argB, veA1) and RJW113.5 (ΔveA::argB, pyrG89). ΔST::AfpyrG indicates that the entire endogenous sterigmatocystin gene cluster was removed from *A. nidulans*. The GMM (glucose minimal medium 1 L containing 50 mL of 20 x nitrate salts, 10 grams of glucose, 1 mL of trace elements) used to grow this auxotrophs RJW256 strain with addition of 1 mL of 0.1% Pyridoxine, 500 mg of uracil and 500 mg of uridine per liter. 20 x nitrate salts (1 L) consists of 120 g $NaNO_3$, 10.4 g KCl, 10.4 g $MgSO_4{\bullet}7H_2O$, 30.4 g $KH_2PO_4$, autoclaved and stored at room temperature.

*Trace Elements* (100mL) contains: 2.20 g $ZnSO_4{\bullet}7H_2O$, 1.10 g $H_3BO_3$, 0.50 g $MnCl_2{\cdot}4\ H_2O$, 0.16 g $FeSO_4{\cdot}7H_2O$, 0.16 g $CoCl_2{\cdot}5H_2O$, 0.16 g $CuSO_4{\cdot}5H2O$, 0.11 g $(NH_4)_6Mo_7O_{24}{\bullet}4H_2O$, 5.00 g $Na_4EDTA$. RJW256 was transformed with individual BGC-FACs and the FAC-vector as shown in Table 2 to produce FAC recombinant strains.

*1.3. FAC Pooling and Illumina-index-sequencing of FAC pools*

Individual FACs were grown in 15 mL tubes. For FAC libraries, each FAC clone of the first 10 plates of FAC libraries of *P. fuscum* (PW2A) and *P. camemberti* (PW2B) were duplicated into a 384-deep-well plate with Terrific Broth (TB) Medium. TB medium: yeast extract, 24 g, tryptone, 20 g, dissolved in 900 mL; Phosphate buffer: 0.17 M $KH_2PO_4$, 0.72 M $K_2HPO_4$, 0.017 M $KH_2PO_4$, 0.072 M $K_2HPO_4$ in 100 mL autoclaved separately, then mixed together after cooling down to room temperature. At this point 8 mL of filter-sterilized 50% Glycerol, (with chloramphenicol to 12.5 ug/mL and arabinose to 0.01%) was added. The duplicated FAC plates were grown in a shaking incubator at 37 °C, 200 rpm for 24 h. Individual FAC-DNAs or FAC-DNA pools from individually grown FAC cells were pooled together using a common alkali-plasmid/BAC-DNA isolation method. Each FAC-DNA or pool was dissolved in 300 uL of 10mM TrisHCl (pH 8.0). In addition to the individual FAC-DNAs, twenty FAC plate-pools (A1-A10, B1-B10), and 40 sub-pools (16 row pools and 24 column pools) were created for each 384-well FAC plate.

Illumina TruSeq indexing library preparation of individual FAC-DNAs or FAC pool DNAs were prepared using Intact Genomics igNext NGS library kits (Intact Genomics). Briefly, FAC-DNA pools or individual FAC-DNAs were sheared to ~500 bp DNA fragments by sonication (sonicator Q800R, Qsonica) and normalized at 25 ng/uL. Approximately 100 ng of fragmented DNA then was end-repaired, dA-tailed, adaptor-ligated and subjected to bead-clean up between steps with Intact Genomics igNext NGS kits for the construction of initial Illumina libraries. Primers of single-indexes (24 indexes available) or dual-indexes (96 x 96 = 9,216 dual combination indexing available) can be used to generate final indexing Illumina libraries with minimal amplification cycles (12~15 cycles). Indexing Illumina libraries were qualified and quantified by both QuBit3 (Lifetech/Invetrogen) and Agilent 2100 Bioanalyzer. We initially used single indexing for 20 FAC pooled DNAs, dual indexing for individual

FACs and late FAC-DNA pools.

We also tested and compared the NGS library kits of *Illumina*, *New England Biolab*, and *Intact Genomics igNext* and found that *Intact Genomics igNext* was equal to or better than the other NGS library kits. Moving forward, all Illumina indexing libraries were prepared with *Intact Genomics igNext NGS kits*. The initial single indexing step was the creation of Illumina libraries of 20 FAC-DNA pools. We had 2 Illumina Miseq runs with v3 chemistry (2 x 300 bp), and generated ~32Gb of sequencing data for individual FACs. We combined the dual-indexing Illumina libraries with other sequencing projects, balanced >1,300 dual-indexing Illumina libraries by an additional MiSeq run (v3, 2 x 75 bp), and generated enough coverage of all libraries (at least 100x) with a HiSeq Xten lane producing 360~400M reads per lane (110~120Gb data output per lane).

The raw MiSeq/HiSeq reads per indexed FAC or FAC pool were imported into an in-house platform based on the Unicycler package (1) for trimming, at a stringency of 0.01% error rate (equivalent to Q score of >40). The trimmed sequences of each indexed (barcoded) FAC or FAC pool were then binned and subjected to independent *de novo assembly,* leading to a single contig (~ 100 kb per FAC), or a set of contigs per FAC pool. When necessary, we incorporated a contemporary all-purpose short read assembler, such as SPAdes (3) to evaluate the performance for both individual FACs and for FAC pools. Assembly metrics based on QUAST and Meta QUAST2 (4) will be evaluated. An auto-assembling, annotation and antiSMASH pipeline (5) was set up for the sequencing data analysis and prediction of the entire set of large SM biosynthetic gene clusters (BGCs) of *P. fuscum* (PW2A) and *P. camemberti* (PW2B), as well as BGCs in individual FACs.

## 2. Tables and figures

**Table S1**. The masses of the crude $CHCl_3$ extracts of each transformant, and the purified masses of each natural product produced by the FAC transformants in fermentation experiments are shown, along with calculated % yields. *Both FACPKS-1L15-1 and FACPKS-2J5-1 produced asperugin A (11) and B (12), but these were detected in the crude extract by analysis of the LC/MS data and were not isolated, purified and quantified.

| Gene Clusters | FAC-Transformants | $CHCl_3$ Extract Mass (mg) | Secondary Metabolites (SM) | Purified Mass of SM | % yield |
|---|---|---|---|---|---|
| A6-C18-S76 | An-2aFACPKS-6B23-2B | 49.1 | asperlin (9) | 30.1 | 61 |
| A6-C5-S7 | An-2aFACPKS-7G5-2B | 48.5 | asperlin (9) | 20.4 | 41 |
| B6-C12-S70 | An-2bFACPKS-9M19-1 | 33.3 | asperlin (9) | 22.1 | 66 |
| B4-C7-S30 | An-2bFACPKS-5A24-3B | 14.8 | strobilurin G (2) | 5.2 | 35 |
| | | | strobilurin F (3) | 2.5 | 17 |
| B8-C3-S6 | An-2bFACPKS-10E3-2B | 131.2 | sequoiamonascin D (4) | 3.5 | 3 |
| | | | sequoiatone A (5) | 2.5 | 2 |
| | | | sequoiatone F (6) | 2.1 | 2 |
| | | | penicillide (7) | 5.1 | 4 |
| | | | dihydroxy-3,5,7-tri-methylisochroman (8) | 31 | 24 |
| B9-C27-S74 | An-2bFACPKS-1K15-2 | 27.8 | asperugin A (11) | 2.1 | 8 |
| | | | asperugin B (12) | 1.8 | 6 |
| B1-C15-S42 | An-2bFACPKS-1L15-1 | 26,2 | asp A (11)* and B (12)* | | |
| B4-C7-S30 | An-2bFACPKS-4K13-2 | 136 | citreohybbridional (1) | 3.1 | 2 |
| | | | asperugin A (11) | 10.2 | 8 |
| | | | asperugin B (12) | 5.3 | 4 |
| B9-C27-S74 | An-2bFACPKS-5L9-1 | 22.2 | asperugin A (11) | 1.1 | 5 |
| | | | asperugin B (12) | 2 | 8 |
| B9-C27-S74 | An-2bFACPKS-2J5-1 | 14.6 | asp A (11)* and B (12)* | | |
| | FAC-*An*HH | 33.2 | farnesol (10) | 2.1 | 8 |
| | | | dihydroxy farnesol (13) | 1.5 | 7 |
| | | | dihydroxy methyl farnesoate (14) | 2.1 | 11 |

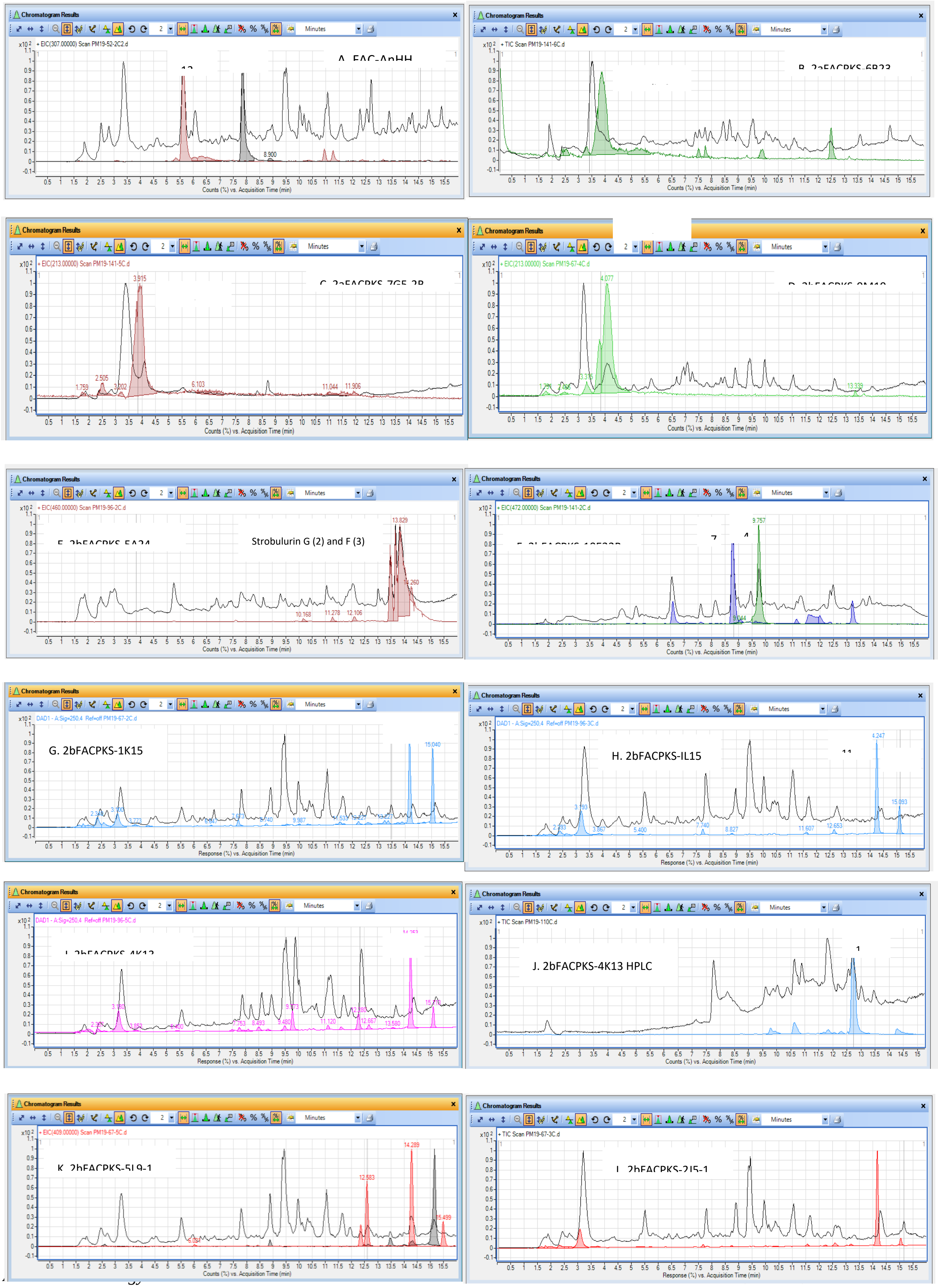
Strobulurin G (2) and F (3)
G. 2bFACPKS-1K15
H. 2bFACPKS-IL15
J. 2bFACPKS-4K13 HPLC

**Figure S1**. Analysis of crude extracts with LC/MS. These are the TIC (Total Ion Chromatogram) traces (A-L) of the crude $CHCl_3$ extracts of the FAC-*An*HH and FAC-Trs used in this study.

TIC is a plot of the sum of all signal intensities of a single scan spectrum against time or scan number. Liquid chromatography/mass spectrometry (LC/MS) experiments were run on Agilent 6520 Q-TOF-LC/MS using a Phenomenex Gemini NX-C18 column. The LC was run in reverse phase gradient mode from 50% $CH_3CN/H_2O$ with 0.1% formic acid to 100% $CH_3CN$ over 15 minutes, then held at 100% $CH_3CN$ for 4 minutes. All solvents used were spectral grade or distilled prior to use. Agilent Mass Hunter Work Station was used for data analysis.

Some of the FAC-Tr LC/MS (TICs) traces differ dramatically from that of the heterologous host (Figure S1A) while some are quite similar, and indicate the presence of overlapping SMs. Some of the most notable differences can be seen between the TIC of the FAC-*An*HH (Figure S1A), and Figure S1B–D, which have large peaks associated with the SM asperlin (9) which has a $[M+1]^+$ of 213 amu. This peak is highlighted in Figure S1B–D, but not present in 1A. Figure S1E has a large peak at 13.8 minutes that correlated to strobilurin G (2) and F (3), which were not detected in the FAC*An*HH or any other FAC-Tr. In Figure S1F, peaks associated with sequoiamonascin (4) and penicillide (7) are clearly seen, but are not detectable in the FAC*An*HH or in other extracts. Several extracts have clear evidence of asperugin A (11) and B (12) in the crude extract, but again, these compounds are not discernible in the heterologous host.

Figure S2a–e NMR Data of Citreohybriddional (1), $C_6D_6$.

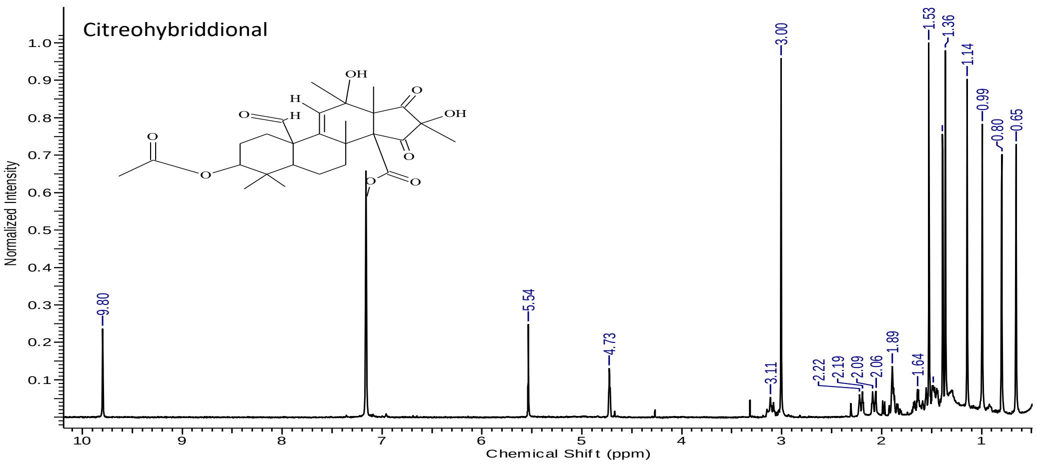


**Supplemental Figure 2a**. $^1H$ NMR of citreohybriddional (1).

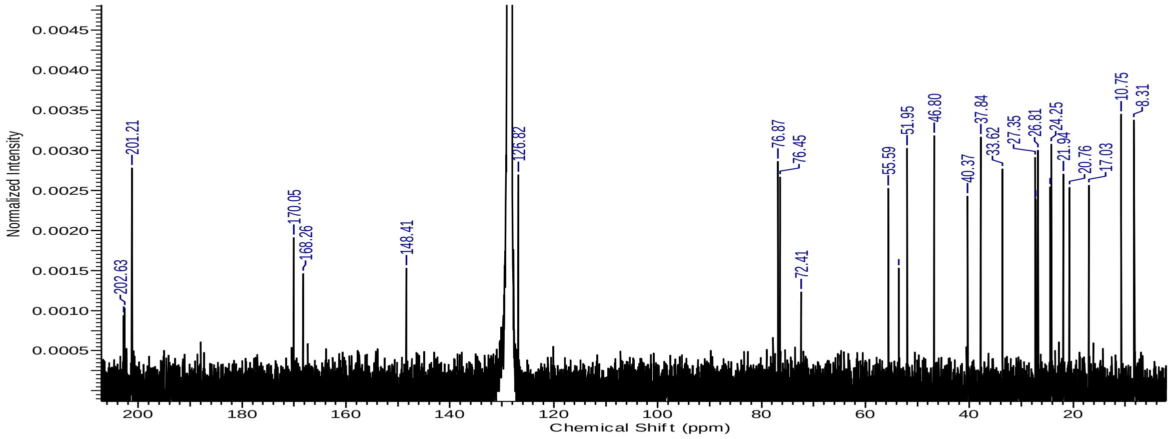


**Figure S2b**. $^{13}C$ NMR of (**1**)

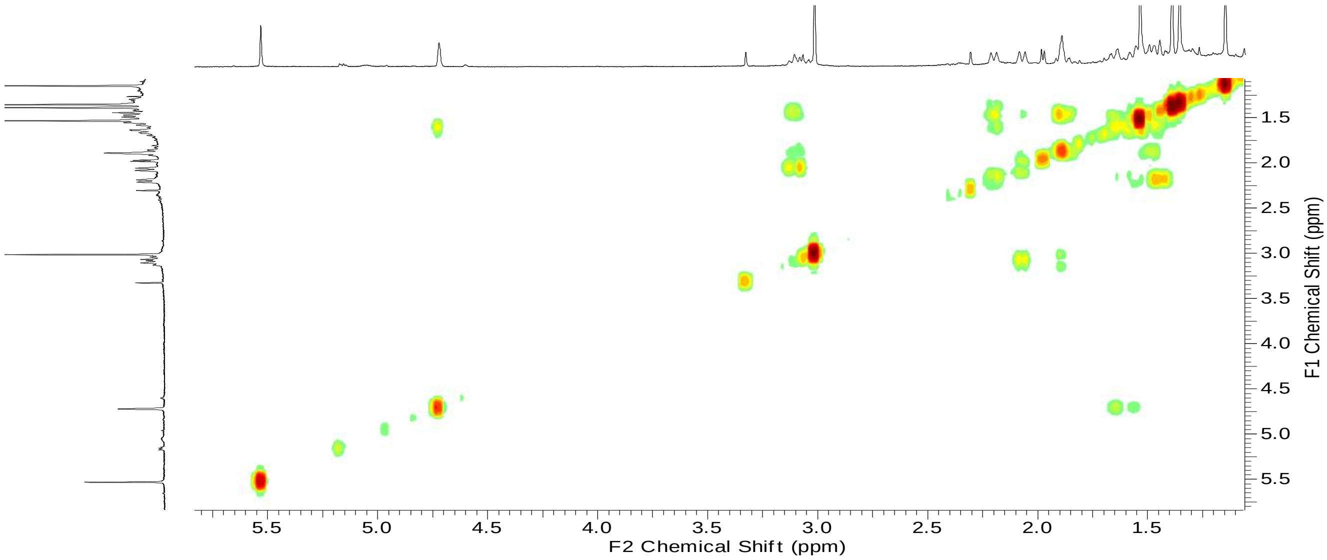


**Figure S2c.** The $^{1}H$-$^{1}H$ COSY of (1) provides proton coupling information which facilitates connectivity.

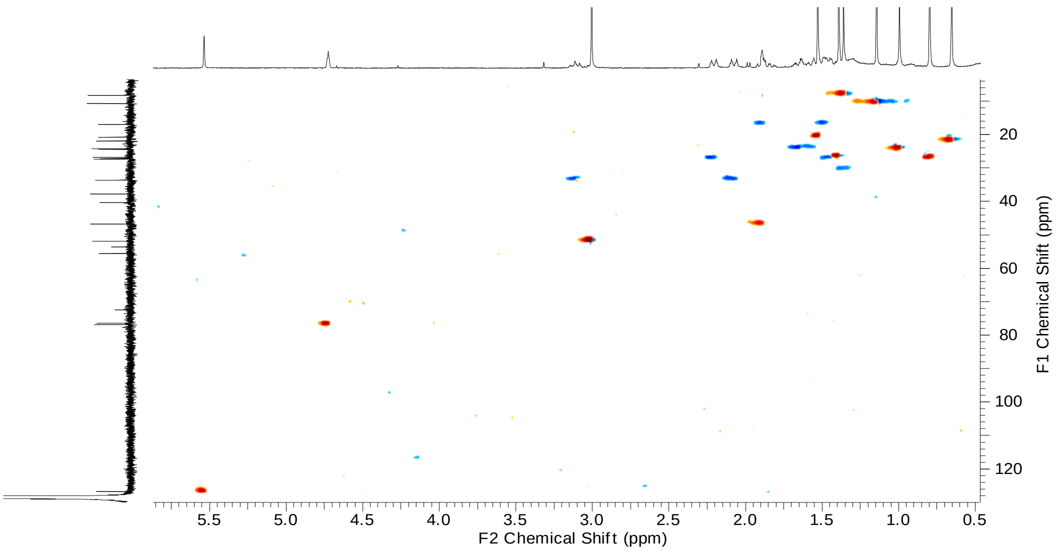


**Figure S2d.** HSQC of (1). $^{1}H$-$^{13}C$ Heteronuclear Single Bond Coherence spectrum allows determination of direct carbon – connectivity. The HSQC experiment is used to determine proton-carbon single bond correlations, where the protons lie along the observed F2 (X) axis and the carbons are along the F1 (Y) axis.

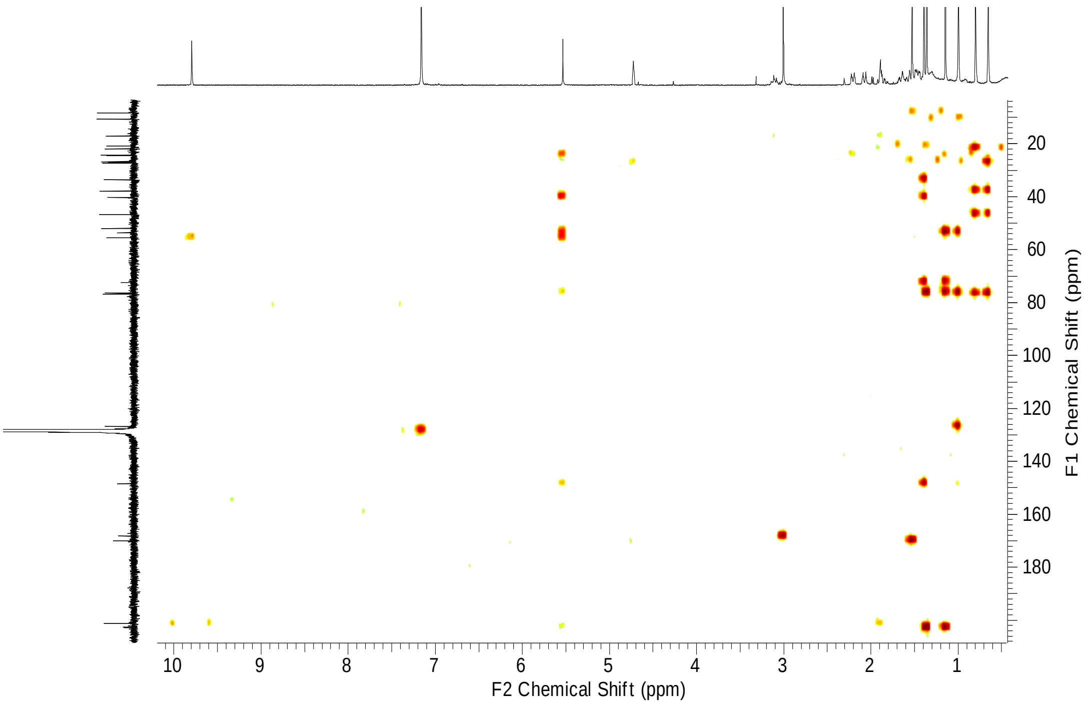


**Figure S2e.** HMBC spectrum of (1). $^{1}H$-$^{13}C$ Homonuclear Multiple Bond Coherence spectrum provides information about 2 – 4 bond coupling between carbons and hydrogens. HMBC spectroscopy correlates $^{1}H$ and $^{13}C$ nuclei through two, three, or sometimes four bonds. Protons lie along the observed F2 (X) axis and the carbons are along the F1 (Y) axis.

Figures S3 a–d. NMR spectra of strobiluring G (2) isolated from 2bFACPKS-5A24-3B. 400 MHz, $CDCl_3$.

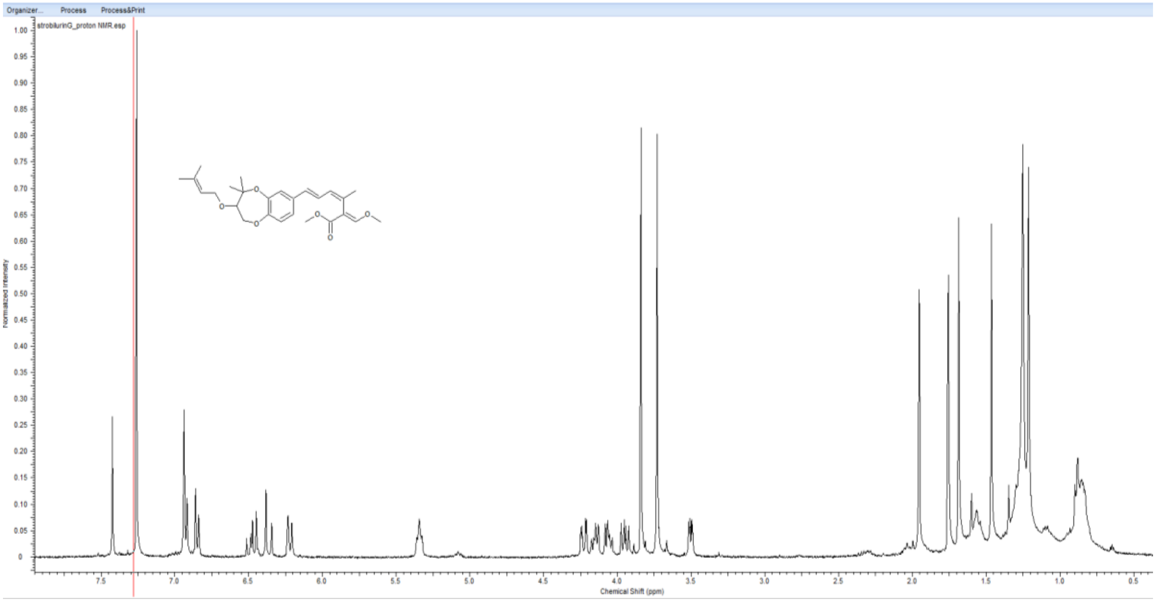


**Figure S3a.** $^{1}$H NMR of (2)

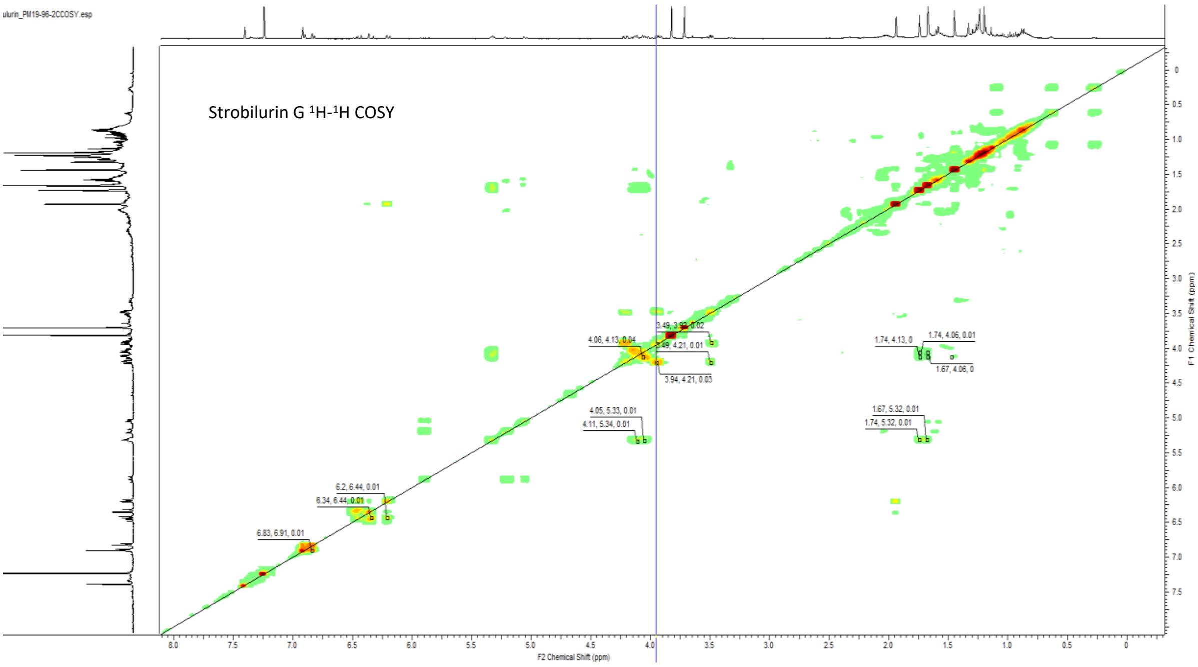


.

**Figure S3b**. $^{1}$H-$^{1}$H COSY (proton-proton correlation) spectrum of strobiluring G (2) isolated from 2bFACPKS-5A24-3B. 400 MHz, CDCl3.

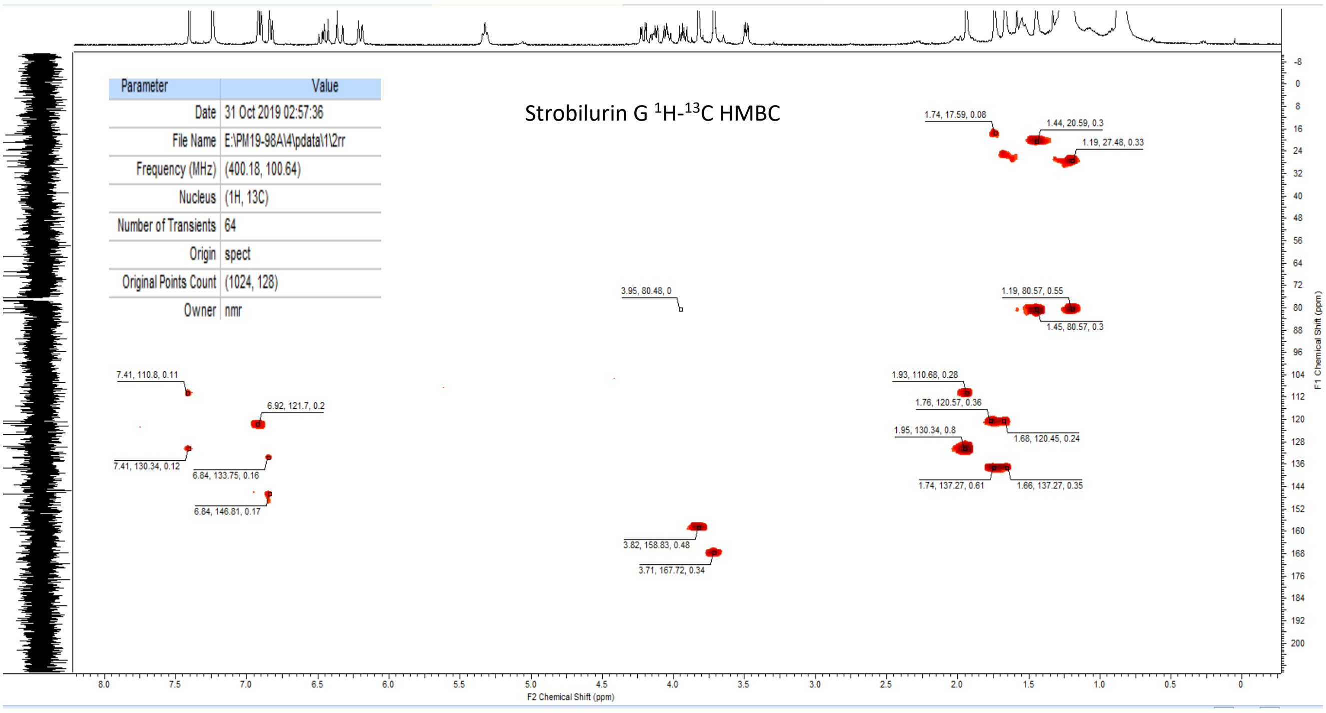


**Figure S3c**. $^{1}$H-$^{13}$C HMBC (Heteronuclear multiple bond correlation) spectrum of strobiluring G isolated from 2bFACPKS-5A24-3B. HMBC spectroscopy correlates $^{1}$H and $^{13}$C nuclei through two, three, or sometimes four bonds. Protons lie along the observed $F_2$ (X) axis and the carbons are along the $F_1$ (Y) axis.

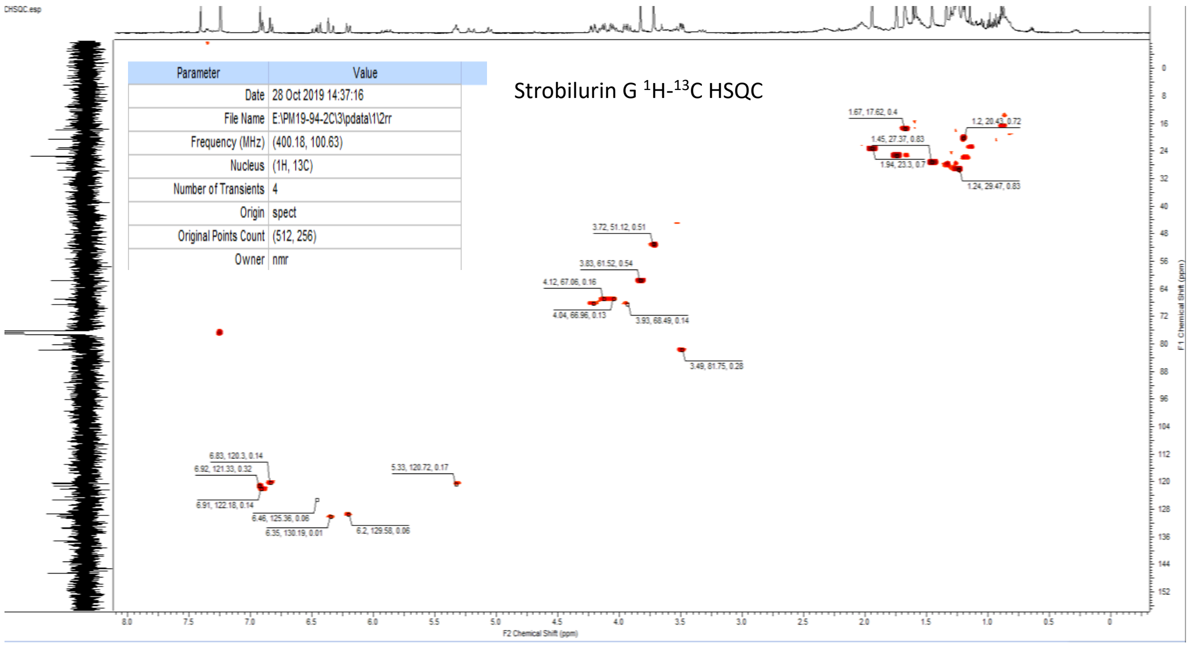


**Figure S3d.** $^{1}$H-$^{13}$C HSQC (Heteronuclear single quantum coherence) spectrum of strobilurin **G** isolated from 2bFACPKS-5A24-3B. The HSQC experiment is used to determine proton-carbon single bond correlations, where the protons lie along the observed $F_2$ (X) axis and the carbons are along the $F_1$ (Y) axis.

**Table S2.** NMR data and structure elucidation of strobilurin G (2).

| Carbon# | Protons | Mult. Coupling in Hz | Carbon (ppm) |
|---|---|---|---|
| 1 | 6.93 | br s | 121.5 |
| 2 | | | 146.7 |
| 3 | | | 150.0 |
| 4 | 6.85 | dd, *J* = 1.14, 8.1 | 120.4 |
| 5 | 6.92 | dd, *J* = 1.14, 8.1 | 122.3 |
| 6 | | | 133.6 |
| 7 | 6.37 | d, *J* = 15.6 | 130.4 |
| 8 | 6.46 | dd, *J* = 10.6, 15.6 | 125.5 |
| 9 | 6.20 | dd, *J* = 1.0, 10.6 | 129.7 |
| 10 | | | 130.6 |
| 11 | | | 110.7 |
| 12 | 7.42 | s | 158.8 |
| 13 | | | 167.7 |
| 14 | 1.94 | s, (3H) | 23.5 |
| 15 | 3.82 | s (3H) | 61.7 |
| 16 | 3.72 | s (3H) | 51.4 |
| 17(a) | 4.23 | dd, *J* = 3.1, 12.4 | 68.6 |
| 17(b) | 3.93 | dd, *J* = 7.9, 12.4 | |
| 18 | 3.48 | dd, *J* = 3.1, 7.9 | 81.8 |
| 19 | | | 80.6 |
| 20 | 1.19 | s | 27.5 |
| 21 | 1.45 | | 20.6 |
| 22a | 4.13 | dd, *J* = 6.8, 11.6 | 67.2 |
| 22b | 4.04 | dd, *J* = 6.8, 11.6 | |
| 23 | 5.32 | tqq, *J* = 6.8, 1.5, 1.5 | 120.8 |
| 24 | | | 137.4 |
| 25 | 1.74 | d (3H), *J* = 1.5 | 25.7 |
| 26 | 1.67 | d (3H), *J* = 1.5 | 17.9 |

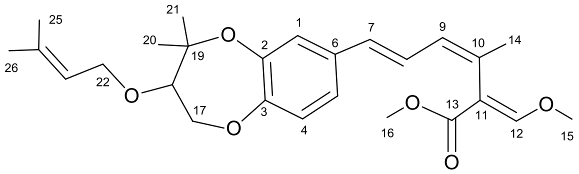

Elucidation of Strobilurin G (2). Strobilurin G (**2**) had a molecular formula of $C_{26}H_{34}O_6$ established by HREIMS ($[M+18]^+$ = 460 amu), with 10 degrees of unsaturation. Inspection of the $^1$H NMR showed evidence of seven allylic/aromatic protons. H-4 and H-5 ($\delta_H$ 6.95 and 6.93) had coupling constants of 8.1 Hz, typical of aromatic protons, so we proposed an aromatic ring system. The large coupling constants 10.6 Hz and 15.6 Hz associated with three protons ($\delta_H$ 6.20, 6.37 and 6.46) suggested two conjugated double bonds. Chemical shift data also indicated three allylic methyls ($\delta_H$ 1.69, 1.76 and 1.96); two methyls deshielded by proximity to a carbonyl group ($\delta_H$ 3.84 and 3.73): and two aliphatic methyls. The carbonyl carbon resonated at $\delta_c$ 167.7, which suggested conjugation with a double bond.

Correlation spectroscopy (HMBC, COSY and HSQC) are powerful tools in elucidation. HSQC enabled the creation of the above chart correlating protons and carbons that were directly coupled to each other. Figure S3e shows proton-proton correlations in the COSY spectrum and proton-carbon correlations in the HMBC spectrum. The HMBC spectrum is optimized for 3-4 bond C-H correlations.

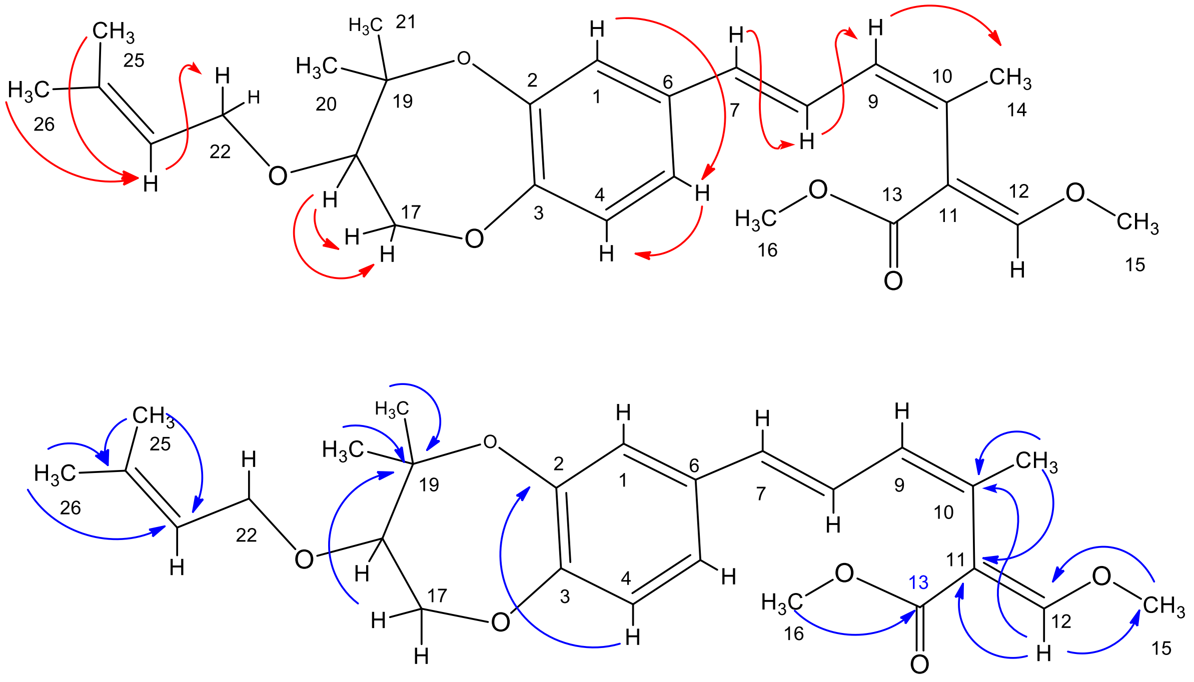

**Figure S4.** Important correlations gleaned from the COSY spectrum (Figure S3b) shown in red and from the HMBC spectrum (Figure S3c) shown in blue facilitated the assemblage of Strobilurin G (2).

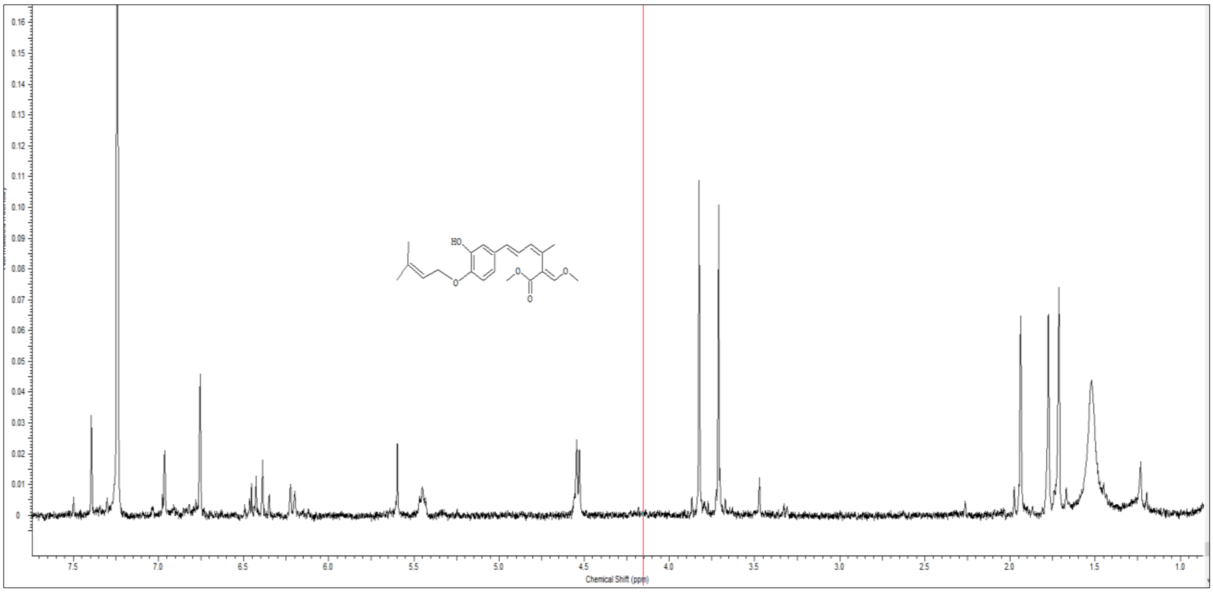


**Figure S5**. $^{1}$H NMR spectrum of strobilurin F (3) isolated from 2bFACPKS-5A24-3B. 400 MHz, $CDCl_3$.

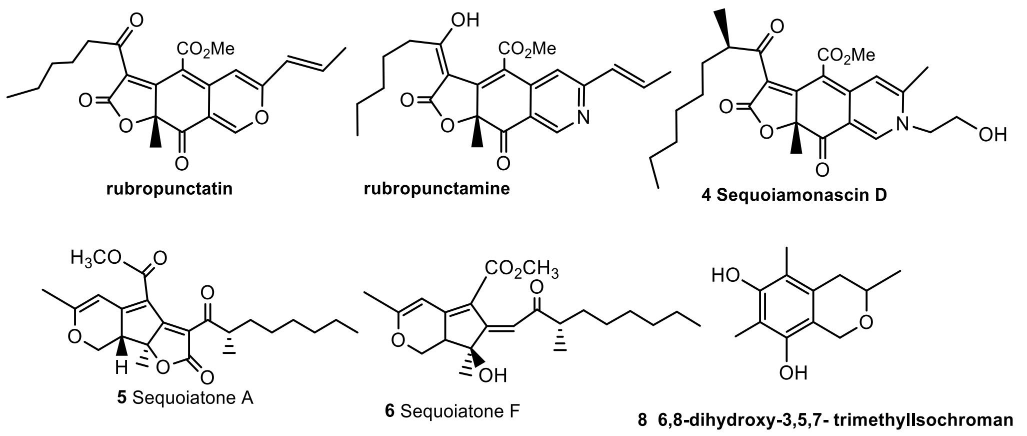


**Figure S6**. Sequoiamonascin D (10) and sequoiatones A (11) and F (12) were synthesized by FAC heterologous expression of FACPKS-10E3-2B. This PKS gene has 29% homologous protein sequence identity to the 2362MpPKS5 gene of the *Monascus pilosus* azaphilone pigment BGC cluster, which is associated with the synthesis of azaphilone type pigments, which includes rubropunctatin and rubropunctamine. Sequoiamonascin D and sequoiatones A and F were originally isolated from the endophyte *Aspergillus parasiticus*, which was harvested from the bark of *Sequoia sempervirens*.

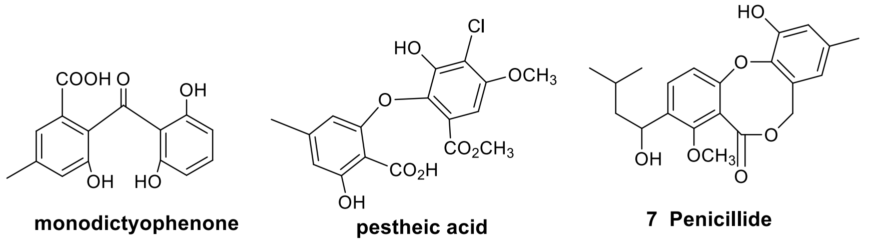


**Figure S7**. The similar structures of penicillide were discovered and elucidated by rapid FAC heterologous expression and related compounds: monodictyophenone, pestheic acid from publications.

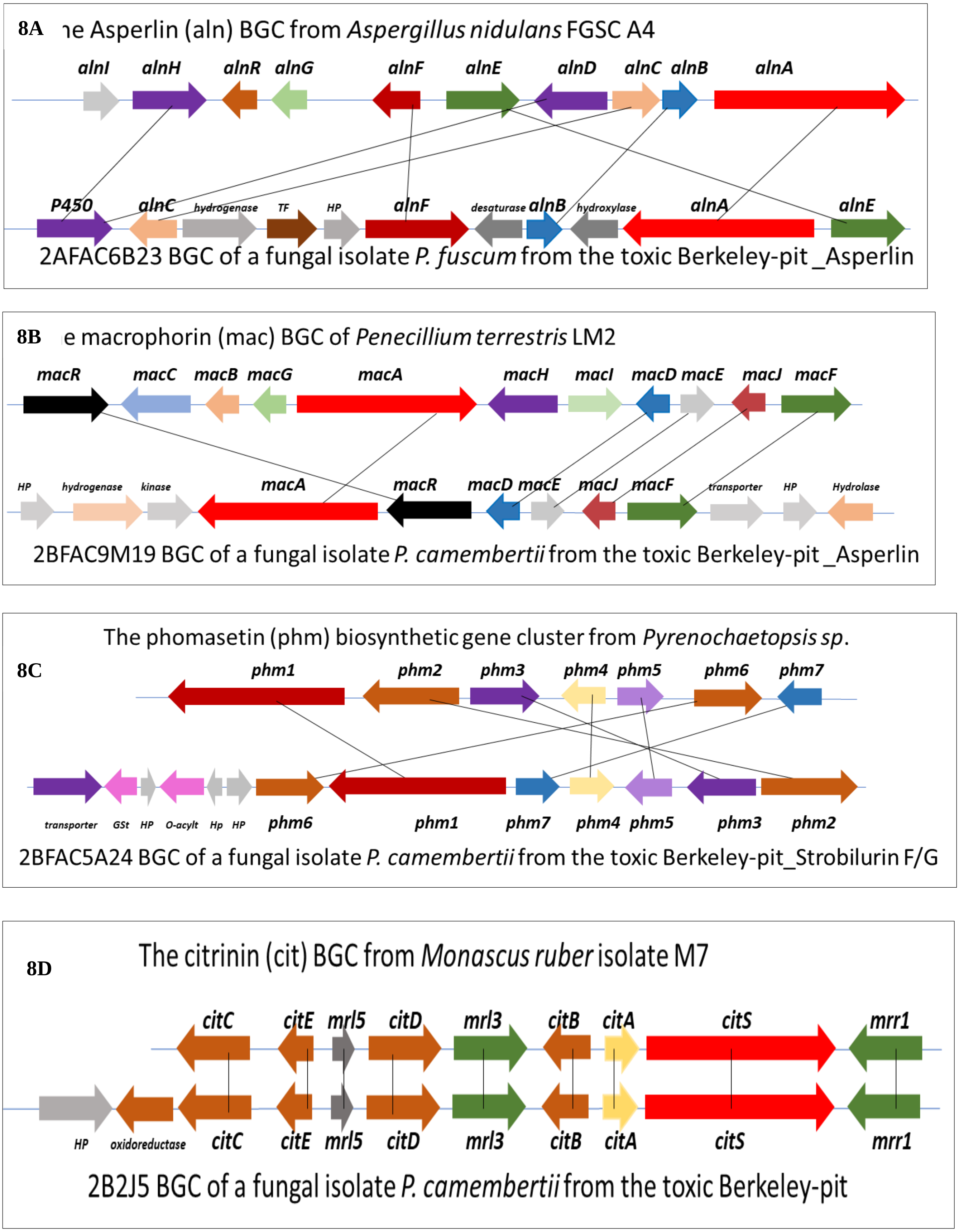
8A
ıe Asperlin (aln) BGC from *Aspergillus nidulans* FGSC A4
alnI alnH alnR alnG alnF alnE alnD alnC alnB alnA
P450 alnC hydrogenase TF HP alnF desaturase alnB hydroxylase alnA alnE
2AFAC6B23 BGC of a fungal isolate *P. fuscum* from the toxic Berkeley-pit _Asperlin
8B
ıe macrophorin (mac) BGC of *Penecillium terrestris* LM2
macR macC macB macG macA macH macI macD macE macJ macF
HP hydrogenase kinase macA macR macD macE macJ macF transporter HP Hydrolase
2BFAC9M19 BGC of a fungal isolate *P. camembertii* from the toxic Berkeley-pit _Asperlin
The phomasetin (phm) biosynthetic gene cluster from *Pyrenochaetopsis sp*.
8C
phm1 phm2 phm3 phm4 phm5 phm6 phm7
transporter GSt HP O-acylt Hp HP phm6 phm1 phm7 phm4 phm5 phm3 phm2
2BFAC5A24 BGC of a fungal isolate *P. camembertii* from the toxic Berkeley-pit_Strobilurin F/G
8D
The citrinin (cit) BGC from *Monascus ruber* isolate M7
citC citE mrl5 citD mrl3 citB citA citS mrr1
HP oxidoreductase citC citE mrl5 citD mrl3 citB citA citS mrr1
2B2J5 BGC of a fungal isolate *P. camembertii* from the toxic Berkeley-pit

**Figure S8**. The homologous BGC alignments of 10 fully sequencing-confirmed BGC-containing FACs of *P. fuscum* (2A) and *P. camembertii* (2B). Three BGC-FACs produced asperlin (9): A. The 2aFAC-6B23 BGC is partially aligned with the silent asperlin (aln) BGC from *A. nidulans* FGSC A4; B. while the 2bFAC9M19 BGC is partially homologous with the macrophorin (mac) from *P. terrestre* isolate LM2; and no homologous BGC with known compound(s) was found for the 2aFAC-7G5 BGC; C. the 2bFAC-5A24 BGC producing Strobilurin F (3) and G (2) is aligned with the entire phomasetin (phm) BGC from *Pyrenochaetopsis* sp.; D. the 2bFAC-2J5 BGC yielding asperugin A (11) and B (12) is homologous to the complete citrinin (cit) BGC from *Monascus ruber* isolate M7E; all gene order is conserved only in these homologous BGCs, besides predicted BGCs between the closely related fungal isolate *P. camembertii* (2B) and *P. expansum*.

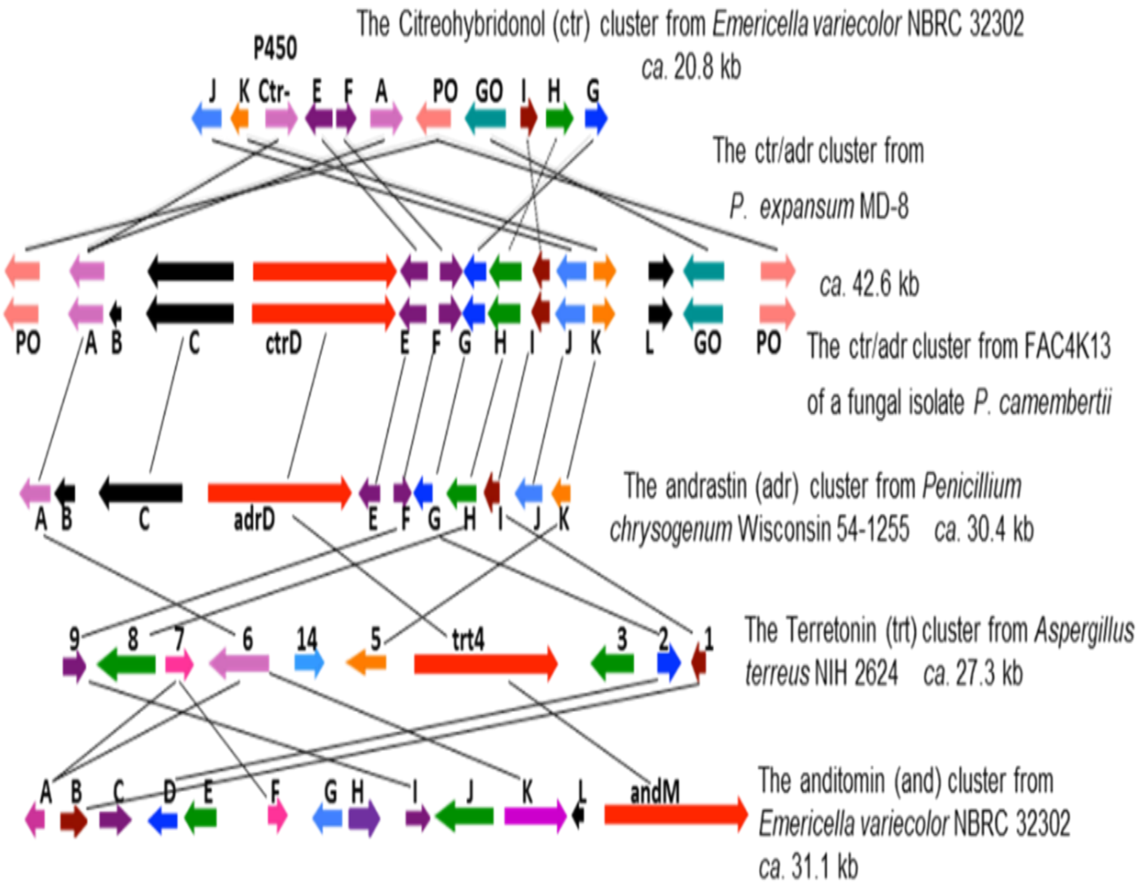


**Figure S9.** The FAC clone 2bFACPKS-4K13 produced citreohybriddional (1), a citreohybriddione analog. The citreohybridones and related meroterpenoids as well as their BGCs have been extensively studied (Supplementary references 6–11)). We have updated the homologous BGC alignment based on the published data (Supplementary references 6–11; Table S2).

Comparison of the 2bFAC4K13 ctr/adr BGC with other homologous BGCs of example fungal multi-ring meroterpenoids published or in NCBI database. Lines indicate the homology between the connected genes. No homologous BGC with known compound(s) in the Genbank database was found

for the rest BGC-containing FACs. Interestingly the BGC of 2bFACPKS-9M19 has homologous protein sequences at 70%~90% identities to 6 genes associated with the 11-member macrophorin (mac) BGC of *Penicillium terrestris* LM254.

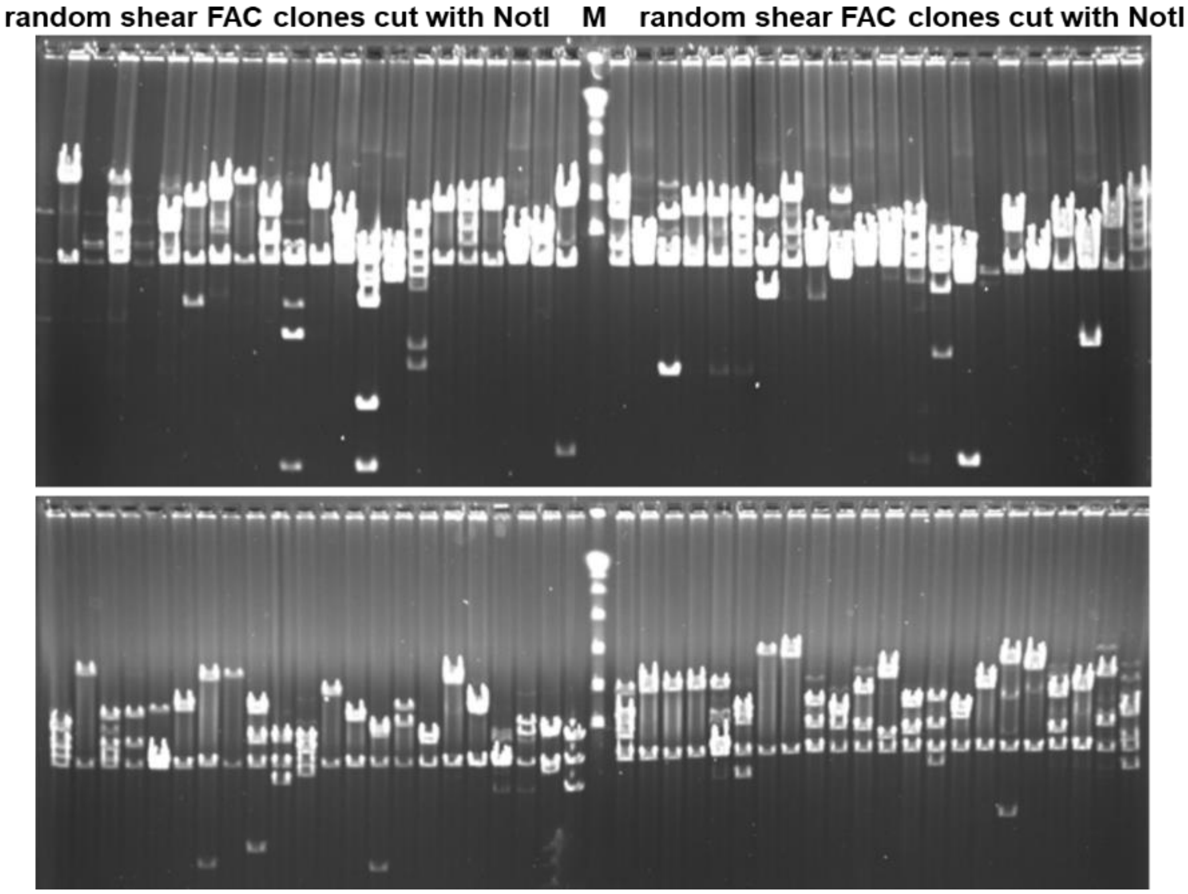


**Figure S10**. The CHEF gels of *E. coli-Aspergillus* shuttle FAC libraries: PW2A (*Penicillium fuscum*) and PW2B (*P. camembertii/clavigerum*), average 110kb inserts were estimated based on >150 randomly picked FAC clones from both libraries. The middle lanes (M) are DNA Lambda ladder Markers, each of the rest of the lanes represents a random FAC clone that was completely digested with NotI.

## Supplementary references

1. Wick RR, Judd LM, Gorrie CL, et al. (2017) Unicycler: resolving bacterial genome assemblies from short and long sequencing reads. *PLoS Comput Biol* 13: e1005595. https://doi.org/10.1371/journal.pcbi.1005595
2. Masella AP, Bartram AK, Truszkowski JM (2012) PANDAseq: paired-end assembler for illumina sequences. *BMC Bioinformatics* 13: 1–7. https://doi.org/10.1186/1471-2105-13-31
3. Bankevich A, Nurk S, Antipov D, et al. (2012) SPAdes: a new genome assembly algorithm and its applications to single-cell sequencing. *J Comput Biol* 19: 455–477. https://doi.org/10.1089/cmb.2012.0021

4. Mikheenko A, Saveliev V, Gurevich A, et al. (2016) MetaQUAST: evaluation of metagenome assemblies. *Bioinformatics* 32: 1088–1090. https://doi.org/10.1093/bioinformatics/btv697
5. Blin K, Shaw S, Steinke K, et al. (2019) antiSMASH 5.0: updates to the secondary metabolite genome mining pipeline. *Nucleic Acids Res* 47: W81–W87. https://doi.org/10.1093/nar/gkz310
6. Guo CJ, Knox BP, Chiang YM, et al. (2012) Molecular genetic characterization of a cluster in A. terreus for biosynthesis of the meroterpenoid terretonin. *Org Lett* 14: 5684–5687. https://doi.org/10.1021/ol302682z
7. Matsuda Y, Awakawa T, Itoh T, et al. (2012) Terretonin biosynthesis requires methylation as essential step for cyclization. *Chem Bio Chem* 13: 1738–1741. https://doi.org/10.1002/cbic.201200369
8. Matsuda Y, Awakawa T, Abe I (2013) Reconstituted biosynthesis of fungal meroterpenoid andrastin A. *Tetrahedron* 69: 8199–8204. https://doi.org/10.1016/j.tet.2013.07.029
9. Matsuda Y, Wakimoto T, Mori T, et al. (2014) Complete biosynthetic pathway of anditomin: nature's sophisticated synthetic route to a complex fungal meroterpenoid. *J Am Chem Soc* 136: 15326–15336. https://doi.org/10.1021/ja508127q
10. Matsuda Y, Iwabuchi T, Wakimoto T, et al. (2015) Uncovering the unusual D-ring construction in terretonin biosynthesis by collaboration of a multifunctional cytochrome P450 and a unique isomerase. *J Am Chem Soc* 137: 3393–3401. https://doi.org/10.1021/jacs.5b00570
11. Matsuda Y, Awakawa T, Mori T, et al. (2016) Unusual chemistries in fungal meroterpenoid biosynthesis. *Curr Opin Chem Biol* 31: 1–7. https://doi.org/10.1016/j.cbpa.2015.11.001

AIMS Press

© 2023 the Author(s), licensee AIMS Press. This is an open access article distributed under the terms of the Creative Commons Attribution License (http://creativecommons.org/licenses/by/4.0)
